# ICAM-1⁺CD51⁺ CAFs drive immunosuppression in colorectal cancer via OPN-triggered chemokine secretion

**DOI:** 10.1186/s12967-026-08642-9

**Published:** 2026-07-17

**Authors:** Jia Liu, Senrui Xue, Yixin Xu, Sicheng Wu, Wenyu Zhao, Xinmiao Li, Nan Hu, Jinmin Sun, Jing Ren

**Affiliations:** 1 Department of Pathology, The Affiliated Hospital of Xuzhou Medical University, Xuzhou Medical University, Xuzhou, 221004 China; 2 Jiangsu Key Laboratory of Brain Disease Bioinformation, Research Center for Biochemistry and Molecular Biology, Xuzhou Medical University, Xuzhou, 221004 China; 3 Department of General Surgery, The Affiliated Hospital of Xuzhou Medical University, Xuzhou, Jiangsu China; 4 Institute of Digestive Diseases, Xuzhou Medical University, Xuzhou, Jiangsu China; 5 Laboratory of Clinical and Experimental Pathology, Department of Pathology, Xuzhou Medical University, Xuzhou, Jiangsu China

**Keywords:** CRC, ICAM-1, CD51, CAFs, Immunosuppression, Immune evasion

## Abstract

**Background:**

Cancer-associated fibroblasts (CAFs) promote colorectal cancer (CRC) progression through immunosuppression, but their functional heterogeneity and specific mechanisms in recruiting myeloid cells remain poorly defined.

**Methods:**

Using functional screening, transcriptomics, and bioinformatics analyses, we identified functionally enriched CAF subsets with differing monocyte-recruiting capacities in CRC. Surface markers were validated via immunofluorescence, immunohistochemistry, and flow cytometry. The role of the identified CAF subset was examined using in vitro co-culture and in vivo tumor models. Animal models were utilized to study the impact of ICAM-1^+^CD51^+^CAFs on CRC tumor growth and immune cell infiltration. Cytokine array profiling, western blotting, and bioinformatics analyses were employed to explore the underlying signaling pathways.

**Results:**

We identified a functionally enriched CAF subset defined by co-expression of ICAM-1 and CD51 (ICAM-1⁺CD51⁺ CAFs), which exhibits potent monocyte-recruiting capacity and drives monocytes to acquire M2-like tumor-associated macrophages (TAMs) and monocytic myeloid-derived suppressor cells (M-MDSCs) associated phenotype and suppressive feature. In clinical CRC samples, the abundance of ICAM-1⁺CD51⁺ CAFs correlated positively with intratumoral TAM infiltration. In vivo validation confirmed that ICAM-1⁺CD51⁺ CAFs promote intratumoral accumulation of both TAMs and MDSCs, thereby accelerating tumor progression. Mechanistically, osteopontin (OPN) engagement of CD51 integrin activated the FAK/p38 MAPK pathway within CAFs, enhancing secretion of key cytokines MCP-3, CXCL5, and IL-6, which orchestrate myeloid cell recruitment and differentiation.

**Conclusion:**

Our study demonstrates that ICAM-1⁺CD51⁺ CAFs regulate cytokine secretion and contribute to myeloid cell recruitment and differentiation in CRC through the OPN/CD51-FAK/p38 MAPK axis. Targeting this CAF subset may represent a promising therapeutic strategy for CRC, which warrants further investigation.

**Supplementary Information:**

The online version contains supplementary material available at 10.1186/s12967-026-08642-9.

## Introduction

 Colorectal cancer (CRC), a prevalent malignancy of the digestive system, ranks as the third most common cancer globally. Over 40% of CRC patients succumb to tumor recurrence and metastasis [1]. Elucidating the molecular mechanisms underlying CRC progression and identifying novel therapeutic targets are therefore critical for diagnosis and treatment. Cancer-associated fibroblasts (CAFs) are pivotal components of the tumor microenvironment (TME), driving CRC metastasis, drug resistance, and immunosuppression [2]. However, recent studies reveal significant heterogeneity in CAFs’ origins, phenotypes, and functions.

Our prior work demonstrated that CAFs with high H19 expression regulate CRC stemness and chemoresistance via exosomes [3]. Su et al. identified CD10^+^GPR77^+^ CAFs that sustain cancer stem cells to promote tumorigenesis and chemotherapy resistance in breast and lung cancers [4]. We further uncovered IL-33^+^ CAFs forming a positive feedback loop with oral squamous carcinoma cells to accelerate progression [5]. Notably, although CAFs-targeted clinical trials are underway, early results have been disappointing. Non-specific CAFs targeting, such as global depletion of FAP^+^ cells or inhibition of general CAFs activation pathways, has been shown to exacerbate tumor aggressiveness in both patients and murine models of pancreatic cancer [6, 7], underscoring the necessity to identify functionally distinct CAFs subsets through specific markers for precise intervention [4]. The current lack of definitive surface markers impedes isolation, functional characterization, and targeted therapy of pro-tumorigenic CAFs subpopulations. Therefore, identifying such subsets by specific surface markers would enable targeted therapeutic intervention.

Understanding CAFs heterogeneity is particularly relevant to immune regulation and therapy resistance. Emerging evidence indicates coexisting immunoinhibitory and immunostimulatory CAFs within the TME [7, 8]. CAFs secrete copious cytokines/chemokines that modulate diverse immune cells—including CD8^+^ T cells, regulatory T cells, and tumor-associated macrophages (TAMs)—to exert dual immunoregulatory effects [2, 9]. As the most abundant immune population in TME, TAMs primarily derive from circulating monocytes and facilitate tumor initiation, invasion, metastasis, and immune escape. Monocytes recruited to tumors differentiate into TAMs or monocytic myeloid-derived suppressor cells (M-MDSCs) under TME cytokine cues [10, 11]. MDSCs, heterogeneous immature cells with potent immunosuppressive capacity, comprise M-MDSC and polymorphonuclear MDSC (PMN-MDSC) subsets and are key mediators of immune evasion [12]. CAFs abundance correlates strongly with TAM infiltration, and their crosstalk collaboratively fuels tumor progression [13, 14]. TAM infiltration levels further associate with CAFs heterogeneity [15, 16]. Single-cell sequencing of prostate CAFs revealed functional diversity in myeloid cell recruitment [17], yet the specific CAFs subsets governing myeloid cell recruitment and the mechanisms remain elusive.

FAP^+^ CAFs in lung adenocarcinoma and squamous carcinoma promote monocyte recruitment via CCL2 [18]. We hypothesized that a specific, marker-defined subset of CAFs is responsible for the heterogeneity in myeloid cell recruitment and actively orchestrates immunosuppression in the CRC tumor microenvironment. Building on these findings, the present study used cell surface markers to identify a functionally enriched, marker-defined CAFs subset, defined by ICAM-1 and CD51 co-expression, that orchestrates the infiltration of TAMs and M-MDSCs in the CRC tumor microenvironment. We further show that these ICAM-1⁺CD51⁺ CAFs actively recruit monocytes and drives monocytes to acquire M2-like TAM/M-MDSC-associated phenotype and suppressive features. Through in vitro and in vivo models, we delineate the functional role of this CAFs subpopulation in shaping immunosuppressive niches. Mechanistically, these CAFs activate the FAK/p38 MAPK pathway via OPN/CD51 integrin signaling, elevating secretion of MCP-3, CXCL5, and IL-6. This cascade may promote monocyte recruitment and their M2-like TAM/M-MDSC-associated phenotype, ultimately facilitating CRC progression.

## Materials and methods

### Cell culture

The mouse colon cancer cell line CT26 was obtained from the American Type Culture Collection (ATCC). Cells were cultured in RPMI-1640 medium (Keygen Biotech, China) supplemented with 10% fetal bovine serum (Life Technologies, USA).

### Isolation and culture of primary CAFs

Human colorectal cancer tissues and adjacent normal tissues were obtained from patients who underwent surgical resection at the Affiliated Hospital of Xuzhou Medical University. The study was conducted in accordance with the Declaration of Helsinki and approved by the Institutional Ethics Committee of the Affiliated Hospital of Xuzhou Medical University (No. XYFY2020-KL125-01). All patients provided written informed consent. Fresh colorectal cancer tissues and corresponding adjacent normal tissues were obtained and immediately washed with PBS containing 20% antibiotics. After washing, the tissues were minced and digested by agitation with a compound enzyme solution (including Collagenase II, Dispase, and Hyaluronidase), with the digestion process monitored closely and terminated as appropriate. The tissue pellet was washed, resuspended in a small volume of DMEM/F12 complete medium supplemented with 12% FBS, and evenly plated in culture dishes. After 3 days, medium was carefully added. One week later, tissue debris and non-adherent cells were washed away. Epithelial cells were removed by trypsinization, leaving behind fibroblasts. The fibroblast population was further purified, cultured, and characterized via additional trypsin digestion. Freshly isolated CAFs (passage 0–1) were used for RNA-seq and initial functional characterization. Cryopreserved CAFs (passage 1) were thawed and cultured to passage 2–3 for functional assays (chemotaxis, co-culture, cytokine arrays).

### Monocyte chemotaxis assay

Monocytes were isolated from peripheral blood using Ficoll density gradient centrifugation. CD14⁺ monocytes were positively selected using magnetic beads (Miltenyi Biotec, human CD14 MicroBeads), and the purity of the isolated monocytes was verified by flow cytometry for subsequent use. Conditioned medium was collected from CAFs (all at passage 2) derived from different patients, centrifuged to remove cell debris, and stored for use. For each CAF isolate, 1 × 10^6^ cells were cultured in 10 mL medium for 48 h; the conditioned medium was then concentrated using Amicon Ultra and normalized to a total protein concentration of 500 µg/mL. The CD14^+^ monocytes ( 1 × 10^4^ cells per well in 100 µL serum-free RPMI-1640) were added to the upper chamber of a Transwell insert (5 μm pore size, Corning). The lower chamber was filled with 200 µL of normalized CAF-conditioned medium. After 4 hours of incubation, the number of migrated CD14^+^ monocytes was quantified by flow cytometry. All assays were performed in triplicate, and migrated cell numbers were normalized to input cell number.

### Immunofluorescence

CRC tissue sections were deparaffinized, subjected to antigen retrieval, and permeabilized with 0.5% Triton X-100. Blocking was performed with 5% goat serum. Sections were incubated with primary antibodies overnight at 4 °C. The specific usage information of the antibodies is listed in the supplementary Table 1. After warming, washing, and incubation with fluorescent secondary antibodies (Alexa Fluor 488-conjugated anti-rabbit, Alexa Fluor 594-conjugated anti-mouse, and Alexa Fluor Plus 647-conjugated anti-goat, 1:800, Invitrogen, USA), nuclei were counterstained with DAPI, and slides were mounted with anti-fade mounting medium.

### Flow cytometric sorting

Fresh CRC tissues were processed into single-cell suspensions using a mixed enzyme digestion method (minced tissues digested with enzymes). The single-cell suspension was stained with fluorescent antibodies for sorting. DAPI⁺ (dead cells), CD45⁺ (leukocytes), CD31⁺ (endothelial cells), and EPCAM⁺ (epithelial cells) were excluded. The sequential gating strategy began with forward scatter versus side scatter to select cells based on size and granularity, followed by singlet discrimination using FSC-A versus FSC-H to exclude doublets. Dead cells were eliminated by gating on DAPI-negative cells. Subsequently, lineage markers were used to remove unwanted populations: CD45-positive leukocytes, CD31-positive endothelial cells, and EPCAM-positive epithelial cells were all excluded from the sorting gate. From the remaining viable, non-lineage cells, the FAP-positive CAFs population was selected. Finally, from within the FAP^+^ gate, ICAM-1⁺CD51⁺ CAFs and ICAM-1⁺CD51⁺ Del CAFs (the CAF fraction depleted of ICAM-1⁺CD51⁺ cells) were sorted as two distinct populations for downstream experiments. Post-sort purity and the expression of ICAM-1, CD51, and α-SMA in the sorted populations were re-verified by flow cytometry to validate target cell isolation. Part of the single-cell suspension was used to analyze the correlation between the number of ICAM-1⁺CD51⁺ CAFs and the numbers of infiltrating MDSCs (CD45⁺HLA-DR⁻CD11b⁺CD33⁺) and M2-TAMs (CD45⁺CD11b⁺CD14⁺CD206⁺) in CRC tissues. The specific usage information of the antibodies is listed in the supplementary Table 1.

### Isolation and culture of primary mouse-derived tumor-associated fibroblasts

Based on the Miltenyi Biotec protocol for mouse CAF isolation [19] and the method by Yang X et al. [20], we optimized the isolation procedure to address issues of low yield and poor viability. Optimizations primarily involved cell inoculation number, use of Matrigel, mouse age, and tumor growth duration. All animal experiments were approved by the Laboratory Animal Ethics Committee of Xuzhou Medical University (No. 202512T048). A total of 1 × 10⁵ CT26 cells mixed 1:1 with Matrigel were subcutaneously injected into the anterior flank of BALB/c mice (9–10 weeks old, male). Tumor growth was monitored. After 45 days, tumor tissues were surgically excised, collecting as much tissue as possible, and washed with PBS containing 20% antibiotics. Tissues were minced and digested with a compound enzyme solution (including Collagenase II, Collagenase IV, DNase I, Dispase, and Hyaluronidase). The tissue pellet was resuspended in a small volume of DMEM/F12 complete medium supplemented with 12% FBS. The tissue suspension was evenly plated in culture dishes. After incubation for 1 h, DMEM/F12 complete medium (with Gibco serum) was gently added to continue culture. After 2 days, medium was carefully replenished. Five days later, tissue debris and non-adherent cells were washed away. Epithelial cells were removed by trypsinization, leaving behind fibroblasts. The fibroblast population was further purified via trypsin digestion, cultured, characterized, and used for subsequent experiments.

### RNA sequencing data

RNA sequencing data of CRC tissues were downloaded from databases, including The Cancer Genome Atlas (TCGA, *n* = 698), Genotype-Tissue Expression (GTEx) database (*n* = 308). Additionally, single-cell RNA sequencing data of CRC were obtained from the Gene Expression Omnibus (GEO) database (GSE178341).

### CAFs-CD14⁺ co-culture system

The CAFs (10⁵) were seeded in the upper chamber of a Transwell insert (0.4 μm pore size, Corning). The lower chamber contained 2 × 10⁵ CD14⁺ monocytes resuspended in RPMI 1640 complete medium. A polycarbonate membrane separated the CAFs from the monocytes while allowing cytokine exchange. The cells were co-cultured for one week, with fresh medium supplemented on day 4. After 7 days, flow cytometry was performed to analyze the expression of CD14, CD11b, CD33, HLA-DR, CD206, CD163, CD15, and CD16 on the surface of monocytes educated by the respective CAF groups. The qPCR was used to detect the expression of genes associated with the immunosuppressive function of MDSCs and M2-TAMs in monocytes educated by the ICAM-1⁺CD51⁺ CAFs and ICAM-1⁺CD51⁺Del CAFs groups. A T cell proliferation suppression assay was conducted to evaluate the immunosuppressive capacity of the educated monocytes.

### In vivo experiments

All animal experiments were approved by the Laboratory Animal Ethics Committee of Xuzhou Medical University (No. 202512T048). The experiment consisted of two groups: ICAM-1⁺CD51⁺ mCAFs and ICAM-1⁺CD51⁺Del mCAFs. CT26 cells were mixed with either ICAM-1⁺CD51⁺ mCAFs or ICAM-1⁺CD51⁺Del mCAFs at a 2:1 ratio, and the mixture was subcutaneously injected into the anterior flank of BALB/c mice (6–7 weeks old, male) to establish a tumor transplantation model. Tumor growth was monitored. The proportions of TAMs (CD45⁺CD11b⁺F4/80⁺), M1-TAMs (CD45⁺CD11b⁺F4/80⁺CD206⁻MHCII⁺), M2-TAMs (CD45⁺CD11b⁺F4/80⁺CD206⁺MHCII⁻), total MDSCs (CD45⁺CD11b⁺Gr1⁺), PMN-MDSCs(CD45^+^CD11b^+^Ly6C^int^Ly6G^+^), M-MDSCs, (CD11b^+^Ly6C^high^Ly6G^−^), and CD8⁺IFN-γ⁺ T cells were analyzed in the harvested tumor tissues. The specific usage information of the antibodies is listed in the supplementary Table 1.

### IHC staining and analysis

Paraffin-embedded tissues were cut into 4-µm sections, deparaffinized in xylene, and rehydrated through graded alcohols. Antigen retrieval was performed using sodium citrate buffer (10 mM, pH 6.0) at 95–100 °C for 15 min in a pressure cooker. After cooling, sections were blocked with 10% goat serum for 30 min at room temperature. Primary antibodies (anti-ICAM-1, anti-CD51, anti-α-SMA; see Supplementary Table 1) were incubated overnight at 4 °C. After PBS washes, HRP-conjugated secondary antibodies were applied for 30 min. DAB substrate was applied for 2–5 min, followed by hematoxylin counterstaining. The expression of ICAM-1, CD51, and α-SMA at the protein level was confirmed based on IHC data obtained from the Human Protein Atlas database. Human CRC tissues were collected from the Pathology Department of the Affiliated Hospital of Xuzhou Medical University.

### T cell proliferation and suppression assay

Isolate CD3⁺ T cells: Harvest mouse spleen, homogenize through a 200-mesh sieve, and lyse red blood cells to obtain a single-cell suspension. Purify CD3⁺ T cells using a mouse CD3⁺ T Cell Isolation Kit (Miltenyi Biotec) according to the manufacturer’s instructions. Verify purity (> 95%) by flow cytometry. Label CD3⁺ T cells with CFSE using a Cell Proliferation Kit (Thermo Fisher). T cells were labeled with 2.5 µM CFSE for 10 min at 37℃, quenched with cold medium, and washed. The educated monocytes (from CAF-monocyte co-culture) were harvested and added to CFSE-labeled T cells at a 1:1 ratio in 96-well plates with anti-CD3/CD28 beads. After 72 h, T-cell proliferation was analyzed by CFSE dilution on a BD FACSCalibur.

### Screening of cytokines secreted by ICAM-1⁺CD51⁺ CAFs and ICAM-1⁺CD51⁺ Del CAFs

Conditioned medium was collected from ICAM-1⁺CD51⁺ CAFs and ICAM-1⁺CD51⁺ Del CAFs. The levels of cytokines in the conditioned medium were analyzed using a human cytokine array. The human cytokine array was performed by Shanghai Universal Biotech Co., Ltd. Protein array membranes (2 per condition) were loaded with 500 µL of cell culture supernatant each. Membranes were blocked with 2 mL of Array Buffer 6 on a shaker for 1 h at room temperature. Samples were diluted to a final volume of 1.5 mL/well using Array Buffers 4 and 5, followed by addition of 15 µL detection antibody per sample and mixing on a shaker for 1 h at room temperature. Samples were then incubated with the array membranes overnight at 4 °C. Post-incubation, membranes were washed 3× with designated Wash Buffer, incubated with HRP-conjugated streptavidin on a shaker for 30 min at room temperature, and washed again 3× with Wash Buffer. Signals were developed by adding 1 mL chemiluminescent substrate per well and visualized using a chemiluminescence imaging system. Data were processed with dedicated array analysis software.

CAFs were seeded at 1 × 10^6^ cells per well in plates and cultured in DMEM/F12 medium supplemented with 10% FBS for 48 h. Conditioned medium was collected and centrifuged at 300 × g for 5 min to remove cell debris, then aliquoted and stored until use. Prior to ELISA, total protein concentration of each sample was determined using a BCA assay, and all samples were normalized to the same protein concentration (500 µg/mL) using culture medium. Commercial ELISA kits (R&D Systems, USA) were used to measure the levels of MCP-3, CXCL5, and IL-6 according to the manufacturer’s instructions.

### Bioinformatics analysis

Gene set enrichment analysis (GSEA) was performed using the clusterProfiler package (v4.6.0) with the MSigDB Hallmark gene sets (h.all.v7.4.symbols). Statistical significance was defined by the following thresholds: |normalized enrichment score (NES)| > 1, nominal p-value < 0.05, and false discovery rate (FDR) < 0.25. Immune infiltration scores were calculated using single-sample GSEA (ssGSEA) as implemented in the R package GSVA (v1.46.0). Correlation analyses between gene expression levels and immune infiltration scores were conducted using the Hmisc package, and the results were visualized with the ggplot2 package.

### Immunoblot analysis

Western blot analysis was performed to detect the protein levels of p38, ERK1/2, and JNK1/2, as well as their phosphorylation status, in ICAM-1⁺CD51⁺ CAFs and ICAM-1⁺CD51⁺Del CAFs. Protein lysates extracted from ICAM-1⁺CD51⁺ CAFs and ICAM-1⁺CD51⁺Del CAFs were quantified using the BCA assay. Proteins were separated via SDS-PAGE electrophoresis, then transferred onto nitrocellulose membranes. Membranes were blocked with bovine serum albumin (BSA) and incubated overnight with primary antibodies. After primary antibody retrieval, membranes were washed and incubated with horseradish peroxidase (HRP)-conjugated secondary antibodies. Following additional washes, protein bands were visualized using SuperSignal™ Chemiluminescent HRP Substrate. Detailed specifications of primary and secondary antibodies are provided in supplementary Table 1.

### Statistical analysis

All data are presented as the mean ± standard deviation (SD). Statistical analyses were performed by GraphPad Prism 7.0 (Graphpad Software Inc) and SPSS 16.0 software (SPSS 16.0, SPSS Inc). Pearson correlation analysis and Chi-square (X^2^) tests were used to perform the correlation analyses. For two-group comparisons, we employed either the Mann-Whitney U test or the two-tailed unpaired Student’s t test, depending on the normality and homogeneity of variance assumptions, and reported Cohen’s d as the effect size. For comparisons involving more than two groups, one-way ANOVA was used, with partial η² reported as the effect size measure, followed by Tukey’s post-hoc test for pairwise comparisons. Pearson correlation analysis was used to assess correlations between continuous variables, with Spearman’s correlation applied for non-normally distributed data. For correlation analyses and multi-group comparisons involving multiple statistical tests, the Benjamini-Hochberg false discovery rate (FDR) method was applied to control for multiple comparisons. For survival analyses, hazard ratios (HR) with 95% confidence intervals (CI) are reported. All experiments were performed with at least three biological replicates. Technical replicates were averaged per biological replicate before statistical analysis; technical replicates were used only to assess assay precision and were not treated as independent observations. Statistical significance was defined as *p* < 0.05.

## Results

### The ICAM-1⁺CD51⁺ CAFs subset exhibits enhanced monocyte-recruiting capacity in vitro

**Fig. 1:**
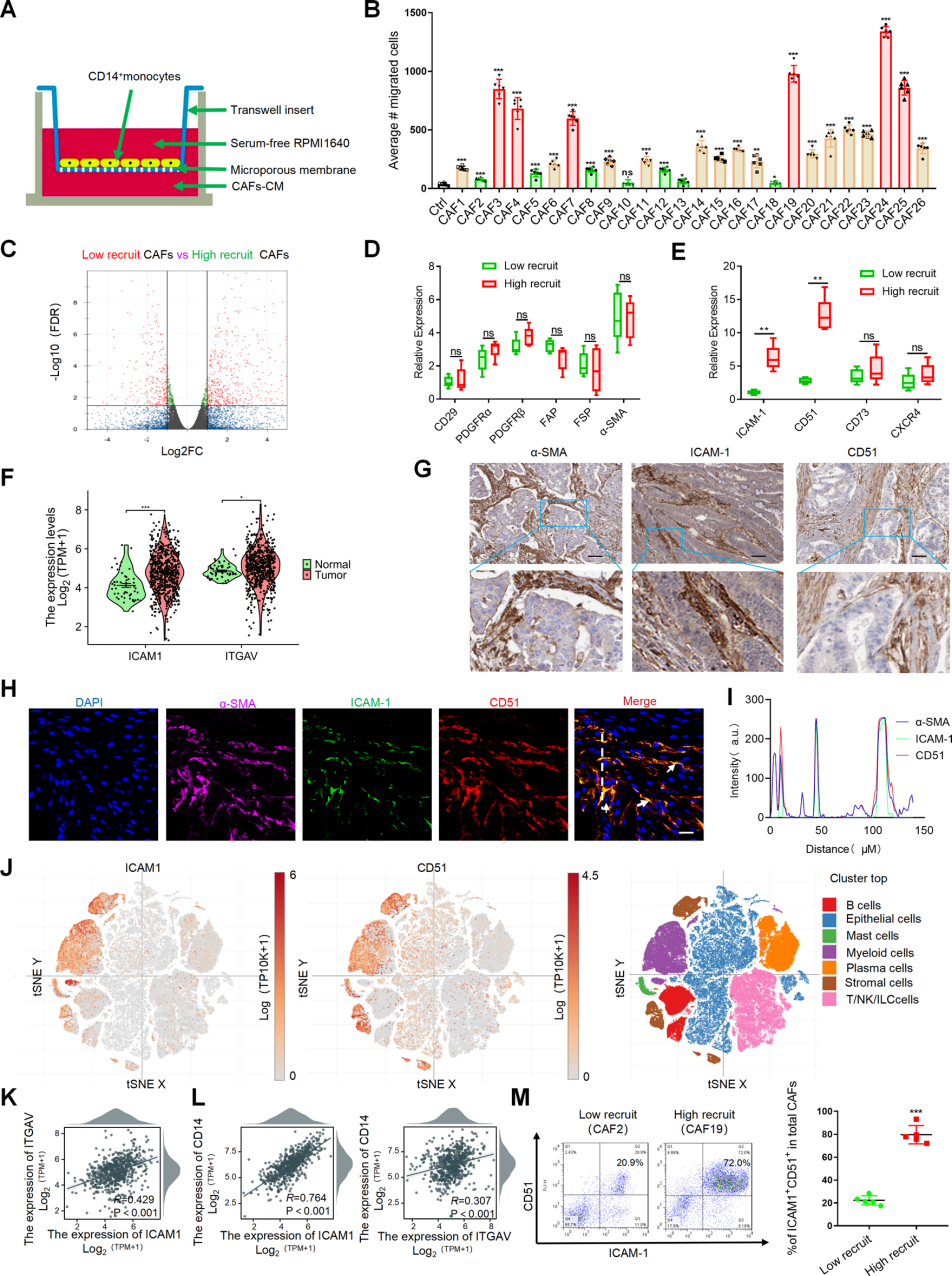
The ICAM-1^+^CD51^+^ CAFs subset correlates with monocyte recruitment capability. (**A**) Schematic diagram of the monocyte chemotaxis assay. CD14^+^ monocytes were resuspended in serum-free RPMI 1640 medium, seeded into the upper chamber, while conditioned medium from CAFs was added to the lower chamber. The number of migrated CD14^+^ monocytes was quantified by flow cytometry. (**B**) Quantification of migrated CD14^+^ monocytes by flow cytometry following chemotaxis assays using conditioned medium from CAFs derived from 26 independent CRC patients. Bars are color-coded by recruitment group: green (low-recruit, *n* = 7), yellow (intermediate-recruit, *n* = 13) and red (high-recruit, *n* = 6). Data represent mean ± SD; each dot represents an individual patient-derived CAF isolate (biological replicate). Statistical significance was determined by one-way ANOVA with Tukey’s post-hoc test (partial η²= 0.53). (**C**) RNA-seq analysis of high monocyte recruitment (CAFs^High recruit^, *n* = 6 biological replicates, each from a different patient) and low recruitment (CAFs^Low recruit^, *n* = 7 biological replicates) CAFs. Heatmap shows differentially expressed genes. DESeq2 was used for differential expression analysis with Benjamini-Hochberg FDR correction (q < 0.05). (**D**) qPCR validation of the expression of α-SMA, FSP-1, CD29, FAP, PDGFRα, and PDGFRβ in the two CAF groups. ns, not significant (two-tailed unpaired Student’s t-test). (**E**) qPCR validation of ICAM-1, CD73, CXCR4, and CD51 expression in high-recruit (*n* = 6 biological replicates) and low-recruit (*n* = 7 biological replicates) CAF groups. Data represent mean ± SD (two-tailed unpaired Student’s t-test). (**F**) Analysis of ICAM-1 and CD51 mRNA levels in colorectal cancer tissues (*n* = 698) versus normal tissues (*n* = 51) from the TCGA database. Data represent mean ± SD. ICAM-1 (95% CI: 0.45–0.97), CD51 (95% CI: 0.035–0.43); Mann-whitney U test. (**G**) Protein expression of ICAM-1 and CD51 predominantly localized in the tumor stroma of colorectal cancer patient tissues. Scale bar (100 μm) (**H**) Immunofluorescence co-localization of ICAM-1, CD51, and α-SMA^+^ stromal cells. Images are representative of *n* = 5 independent patient samples (biological replicates). scale bars (50 μm) (**I**) Fluorescence intensity profile along the indicated line (white dashed line) in the merged image. The X-axis represents the distance (in µm) and the Y-axis represents the normalized fluorescence intensity (arbitrary units, A.U.) of α-SMA, ICAM-1 and CD51. The overlapping peaks indicate regions of co-localization between the proteins. (**J**) Expression analysis of ICAM-1 and CD51 in single-cell RNA-seq data from colorectal cancer. (**K**) Correlation analysis of ICAM-1 and CD51 (ITGAV) expression levels in CRC samples from the TCGA database (*n* = 698). Correlation coefficient *r* = 0.429 (95% CI: 0.34–0.47); q < 0.001. (**L**) Analysis of the correlation between ICAM-1/CD51 and CD14 levels in CRC samples from the TCGA database. ICAM-1 vs. CD14: Correlation coefficient *r* = 0.764 (95% CI: 0.73–0.79); q < 0.001; CD51 vs. CD14: Correlation coefficient *r* = 0.307 (95% CI: 0.18–0.33); q < 0.001; (**M**) Flow cytometric analysis of the proportion of the ICAM-1^+^CD51^+^ CAFs subset in CAFs^high recruit^ and CAFs^Low recruit^ groups (two-tailed unpaired Student’s t-test). Data represent mean ± SD. Cohen’s d = 9.02 (95% CI: 4.91–13.15); two-tailed unpaired Student’s t-test. All p-values are two-sided; q-values (FDR-corrected) are reported where multiple comparisons were performed; technical replicates were averaged per biological replicate before statistical analysis. ns, not significant; * P < 0.05, **P < 0.01, ***P < 0.001

During the preliminary phase of this study, leveraging our well-established protocol for the isolation, cultivation, and identification of primary colorectal cancer (CRC)-derived fibroblasts [3], we isolated and cultured primary CAFs from tumor tissues of 26 CRC patients. Our initial investigations into the monocyte-recruiting capacity of CAFs revealed substantial heterogeneity between CAFs derived from different patients (all at passages 2–3) regarding their ability to recruit monocytes (Fig. 1 A-B). Based on the distribution of chemotaxis indices across 26 patient-derived CAF isolates, CAFs were stratified into three groups: low-recruit (CAFs^Low recruit^, chemotaxis index < 4.3), intermediate-recruit (4.3 ≤ index ≤ 15.6), and high-recruit (CAFs^High recruit^, index > 15.6). Transcriptome sequencing was subsequently conducted on CAFs^High recruit^ and CAFs^Low recruit^group (Fig. 1 C). Analysis indicated no significant differences in the expression of established CAF markers—such as α-SMA, FSP, CD29, FAP, PDGFRα, and PDGFRβ—between the high- and low-recruitment groups (Fig. 1D), suggesting that conventional CAF markers are not predictive of monocyte-recruitment capacity. We systematically screened candidate membrane protein-encoding genes for their expression differences between high-and low-recruit CAFs. Focusing on genes with the most marked intergroup variation, we examined those encoding surface proteins and cross-referenced the Human Protein Atlas for stromal expression patterns in CRC. This approach identified ICAM-1 and ITGAV (encoding CD51) as the top candidates, whose mRNA levels differed markedly between the two CAF groups (Fig. 1D and E).

Analysis of mRNA levels from TCGA colorectal cancer datasets confirmed that both ICAM-1 and CD51 are highly expressed in CRC tissues (Fig. 1 F). ICAM-1 and CD51 expressions in CRC with different clinicopathological characteristics were analyzed based on the TCGA data. Overexpression of ICAM-1 was significantly correlated with gender and histological type (Table 1). Overexpression of CD51 was significantly correlated with residual tumor, lymphatic invasion and neoplasm type (Table 2). Immunohistochemistry further revealed that, similar to the conventional CAF marker α-SMA, both ICAM-1 and CD51 proteins localize predominantly within the tumor stroma of CRC patient tissues (Fig. 1G). Immunofluorescence co-localization assays confirmed that ICAM-1 and CD51 are co-expressed in α-SMA-positive stromal cells (Fig. 1H and I). Single-cell RNA sequencing data from CRC tissues indicated that both ICAM-1 and CD51 are expressed within a subset of stromal cells (Fig. 1J). Analysis of TCGA data also demonstrated a significant positive correlation between ICAM-1 and CD51 mRNA expression in CRC specimens (Fig. 1K). In addition, the expression levels of ICAM-1 and CD51 each showed strong positive correlations with markers of monocytes (CD14) and myeloid-derived suppressor cells (MDSCs, CD33) in CRC tissues (Fig. 1L and Supplementary Fig. 1). Importantly, we observed a notably higher proportion of ICAM-1^+^CD51^+^ CAFs in the CAFs^high recruit^ group, which exhibits stronger monocyte recruitment capability (Fig. 1M).

**Table 1:** Association between ICAM1 mRNA expression and the clinical parameters of CRC patients in TCGA

Characteristics	Low expression of ICAM1	High expression of ICAM1	*P* value
n	322	322	
Perineural invasion, n (%)			0.058
No	86 (36.6%)	89 (37.9%)	
Yes	21 (8.9%)	39 (16.6%)	
CEA level, n (%)			0.194
<= 5	115 (27.7%)	146 (35.2%)	
> 5	78 (18.8%)	76 (18.3%)	
Lymphatic invasion, n (%)			0.894
No	170 (29.2%)	180 (30.9%)	
Yes	114 (19.6%)	118 (20.3%)	
Age, n (%)			0.750
<= 65	140 (21.7%)	136 (21.1%)	
> 65	182 (28.3%)	186 (28.9%)	
Gender, n (%)			**0.009**
Female	167 (25.9%)	134 (20.8%)	
Male	155 (24.1%)	188 (29.2%)	
Histological type, n (%)			**0.022**
Adenocarcinoma	286 (45.2%)	264 (41.7%)	
Mucinous adenocarcinoma	32 (5.1%)	51 (8.1%)	

**Table 2:** Association between ITGAV (CD51) mRNA expression and the clinical parameters of CRC patients in TCGA

Characteristics	Low expression of ITGAV	High expression of ITGAV	*P* value
n	322	322	
Pathologic stage, n (%)			0.745
Stage I	57 (9.1%)	54 (8.7%)	
Stage II	116 (18.6%)	122 (19.6%)	
Stage III	93 (14.9%)	91 (14.6%)	
Stage IV	50 (8%)	40 (6.4%)	
Histological type, n (%)			**0.031**
Adenocarcinoma	282 (44.5%)	268 (42.3%)	
Mucinous adenocarcinoma	32 (5.1%)	51 (8.1%)	
Residual tumor, n (%)			**0.034**
R0	237 (46.5%)	231 (45.3%)	
R1	4 (0.8%)	2 (0.4%)	
R2	26 (5.1%)	10 (2%)	
Lymphatic invasion, n (%)			**0.046**
No	162 (27.8%)	188 (32.3%)	
Yes	127 (21.8%)	105 (18%)	
Neoplasm type, n (%)			**0.007**
Colon adenocarcinoma	224 (34.8%)	254 (39.4%)	
Rectum adenocarcinoma	98 (15.2%)	68 (10.6%)	
Age, n (%)			0.080
<= 65	127 (19.7%)	149 (23.1%)	
> 65	195 (30.3%)	173 (26.9%)	
Gender, n (%)			0.236
Female	143 (22.2%)	158 (24.5%)	
Male	179 (27.8%)	164 (25.5%)	

### ICAM-1^+^CD51^+^ CAFs exhibit strong recruitment and induction-differentiation capabilities for monocytes

**Fig. 2:**
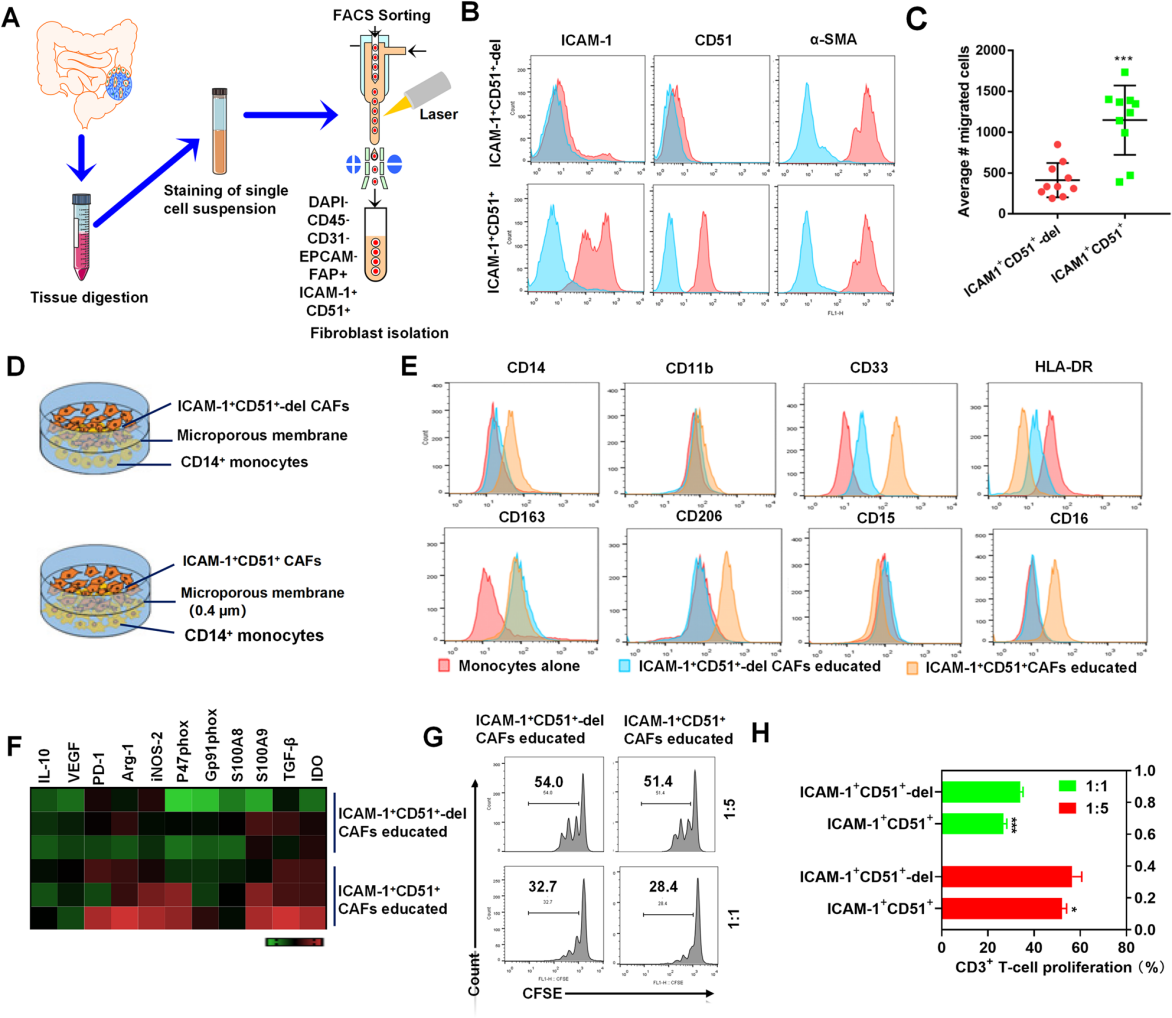
ICAM-1^+^CD51^+^ CAFs exhibit strong recruitment and induction-differentiation capabilities for monocytes. (**A**) Flowchart for the isolation of ICAM-1^+^CD51^+^ CAFs from CRC tissue samples. The tissue is processed into a single-cell suspension, followed by fluorescent labeling and flow cytometry sorting. DAPI^+^ (dead cells), CD45^+^ (leukocytes), CD31^+^ (endothelial cells), and EPCAM^+^ (epithelial cells) are excluded, and ICAM-1^+^CD51^+^ CAFs and ICAM-1^+^CD51^+^ Del CAFs are sorted from FAP^+^ cells. (**B**) Flow cytometry analysis of the expression of ICAM-1, CD51, and α-smooth muscle actin (α-SMA) in ICAM-1^+^CD51^+^ CAFs and ICAM-1^+^CD51^+^ Del CAFs. Data are representative of n=10 independent patient-derived CAF isolates (biological replicates). (**C**) Monocyte recruitment assay using conditioned media from ICAM-1^+^CD51^+^ CAFs and ICAM-1^+^CD51^+^ Del CAFs. The number of migrated CD14^+^ monocytes is counted and statistically analyzed by flow cytometry. Data are representative of n=10 independent patient-derived CAFs isolates (biological replicates). Data represent mean ± SD. Two-tailed unpaired Student's t-test was used. (**D**) Schematic diagram of the CAFs-CD14^+^ monocyte co-culture system, where two groups of CAFs are co-cultured with CD14^+^ monocytes for 7 days, respectively. (**E**) After co-culture, the expression of CD14, CD11b, CD33, HLA-DR, CD206, CD163, CD15, and CD16 on the surface of monocytes before and after education is detected. Data are representative of n=5 independent experiments. (**F**) qRT-PCR analysis of genes associated with immunosuppressive functions of MDSCs and M2-type TAMs in monocytes educated by ICAM-1⁺CD51⁺ CAFs (n=3 biological replicates) versus ICAM-1⁺CD51⁺ Del CAFs (n=3 biological replicates). Data represent mean ± SD of three technical replicates per biological replicate. (**G-H**) T-cell suppression assay to detect the immunosuppressive capacity of educated monocytes. Data represent mean ± SD from n=4 independent biological replicates (two-tailed unpaired Student's t-test). P<0.05, ***P<0.001

To further investigate the functions of the ICAM-1^+^CD51^+^ CAFs subset, we performed flow cytometry sorting to isolate ICAM-1^+^CD51^+^ CAFs and CAFs subsets depleted of ICAM-1^+^CD51^+^ (ICAM-1^+^CD51^+^ Del CAFs) from fresh CRC patient samples. Sorted ICAM-1⁺CD51⁺ CAFs were subjected to cryopreservation, thawing, and re-culture, and were also maintained in culture for more than two weeks. Flow cytometric re-analysis revealed that the sorted population retained high double-positive expression (> 95%, Supplementary Fig. 2), indicating relative phenotypic stability in vitro. We verified that both ICAM-1^+^CD51^+^ CAFs and ICAM-1^+^CD51^+^ Del CAFs were α-smooth muscle actin (α-SMA)-positive cells (Fig. 2A-B). Through monocyte chemotaxis assays, we found that the recruitment capacity of ICAM-1^+^CD51^+^ CAFs for monocytes was significantly stronger than that of ICAM-1^+^CD51^+^ Del CAFs (Fig. 2C). Given that, under pathological tumor conditions, recruited monocytes can acquire the associated phenotype of TAMs, dendritic cells (DCs), and MDSCs, we established a CAFs-CD14^+^ co-culture system (Fig. 2D). The results showed that the levels of markers for M2-type TAMs (CD206 and CD163) and M-MDSCs (CD33, CD14, and CD16) on the surface of monocytes educated by ICAM-1^+^CD51^+^ CAFs were significantly elevated. Meanwhile, the expression level of HLA-DR on the surface of monocytes decreased after education by ICAM-1^+^CD51^+^ CAFs. However, we observed no significant changes in the levels of myeloid cell markers (CD11b) and granulocyte-like markers (CD15) (Fig. 2E). These findings indicate that ICAM-1^+^CD51^+^ CAFs can drive monocytes to acquire M2-like TAM/M-MDSC-associated phenotypic and suppressive features.

**Fig. 3:**
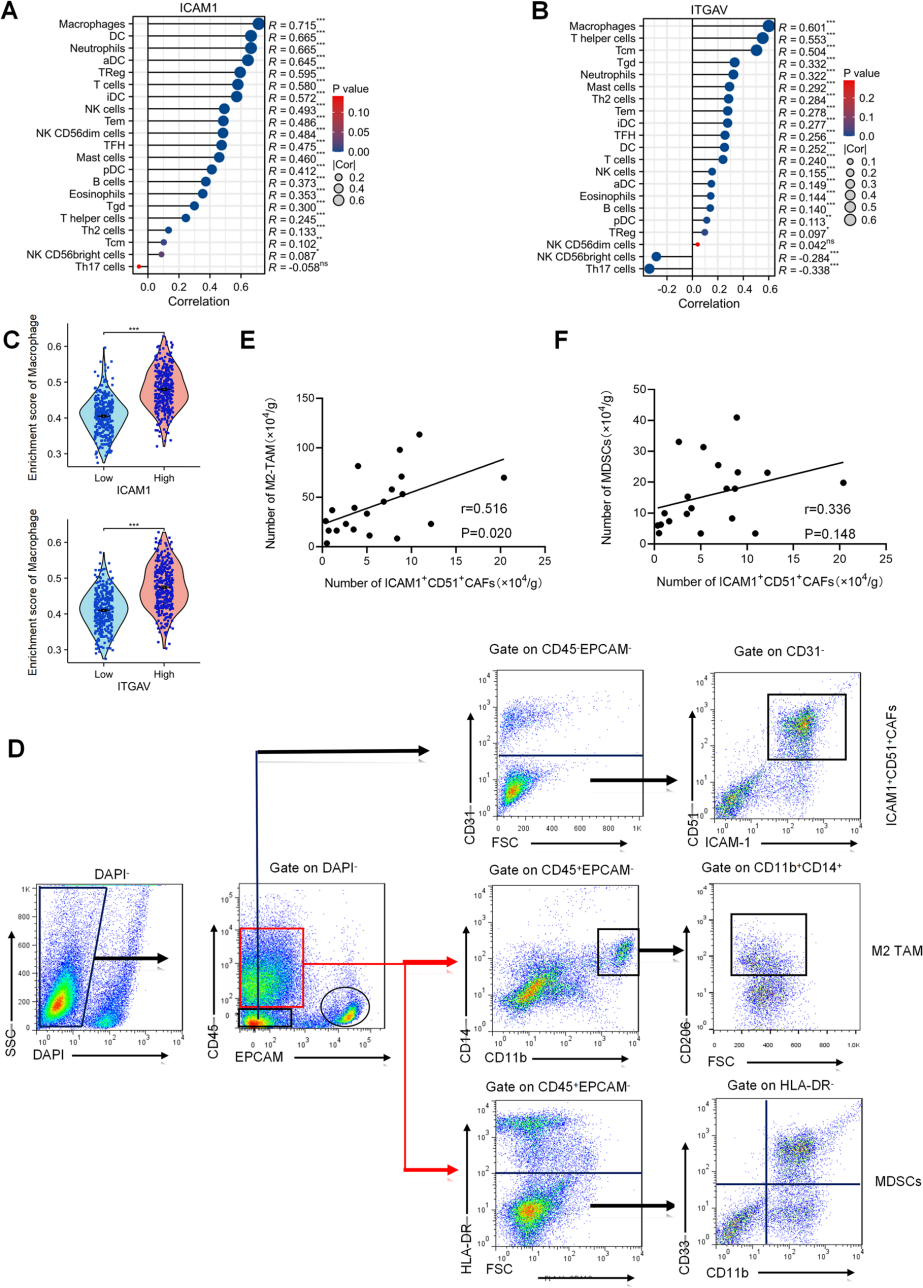
The abundance of ICAM-1^+^CD51^+^ CAFs in CRC patient tissues is correlated with the infiltration of TAMs. (**A**) Lollipop plots of the correlation analysis between the ICAM-1 level and different immune cells in CRC from TCGA. The size of dots represents the value of Spearman’s R. (**B**) Lollipop plots of the correlation analysis between the ITGAV level and different immune cells in CRC from TCGA. The size of dots represents the value of Spearman’s R. (**C**) The macrophage infiltration scores in high expressions of ICAM-1 and CD51 compared to those with low expressions of ICAM-1 and CD51. Mann-whitney U test was used. (**D**) The main gating strategies used in flow cytometry analysis for ICAM-1^+^CD51^+^ CAFs, TAMs, and MDSCs. (**E**) Analysis of the correlation between the number of ICAM-1^+^CD51^+^ CAFs and the number of infiltrating M2-type TAMs (CD45^+^CD11b^+^CD14^+^CD206^+^) in CRC tissues. Data are representative of *n* = 20 independent patient samples. Pearman’s *r* = 0.516 (95% CI: 0.108–0.787), q = 0.019 (Benjamini-Hochberg FDR correction). (**F**) Analysis of the correlation between the number of ICAM-1^+^CD51^+^ CAFs and the number of infiltrating MDSCs (CD45^+^HLADR-CD11b^+^CD33^+^) in CRC tissues. Data are representative of *n* = 20 independent patient samples. Pearman’s *r* = 0.336 (95% CI: -0.119-0.678), q = 0.148 (Benjamini-Hochberg FDR correction). Technical replicates were averaged per biological replicate before analysis,***P < 0.001

Additionally, we found that monocytes, after being educated by ICAM-1^+^CD51^+^ CAFs, exhibited upregulated expression of genes associated with the immunosuppressive functions of common MDSCs and M2-type TAMs (Fig. 2F) and significantly inhibited T-cell proliferation (Fig. 2G-H). Together, these findings demonstrate that ICAM-1^+^CD51^+^ CAFs not only drives monocytes to acquire M2-like TAM/M-MDSC-associated phenotype but also augment the immunosuppressive activity of these myeloid cells. These results demonstrate that ICAM-1^+^CD51^+^ CAFs possess a strong ability to drives monocytes to acquire M2-like TAM/M-MDSC-associated phenotype and enhance their immunosuppressive functions.

### The abundance of ICAM-1^**+**^CD51^**+**^ CAFs in CRC patient tissues is correlated with the infiltration of TAMs

We analyzed the relationship between ICAM-1 and ITGAV expression and immune infiltration to explore the role of ICAM-1^+^CD51^+^CAFs in the immune regulation of CRC. Notably, both the ICAM-1 and ITGAV expression was strong positively correlated with tumor infiltration by macrophages (Fig. 3A-B). The CRC tumor tissues with high expression of ICAM-1 and CD51 showed a significantly higher macrophage infiltration score compared to those with low expression of ICAM-1 and CD51 (Fig. 3C). We performed additional bioinformatics analyses, which revealed that both ICAM-1 and ITGAV showed significant positive correlations with CD8⁺ T-cell exhaustion markers incuding PD1（PDCD1）, TIGIT, TIM-3 (HAVCR2), CTLA4 and 2B4 (CD244) and as well as Treg markers FOXP3 (Supplementary Figure. 3A-B). Flow cytometric analysis of CRC patient samples revealed that the number of ICAM-1^+^CD51^+^ CAFs was positively correlated with the number of infiltrating M2-type TAMs (CD45^+^CD11b^+^CD14^+^CD206^+^) (Fig. 3D-E). In contrast, no significant correlation was observed between the number of ICAM-1^+^CD51^+^CAFs and the number of infiltrating MDSCs (CD45^+^HLADR^-^CD11b^+^CD33^+^) (Fig. 3D-F).

### ICAM-1^+^CD51^+^ CAFs increase intratumoral infiltration of TAMs and MDSCs and promote tumor growth

**Fig. 4:**
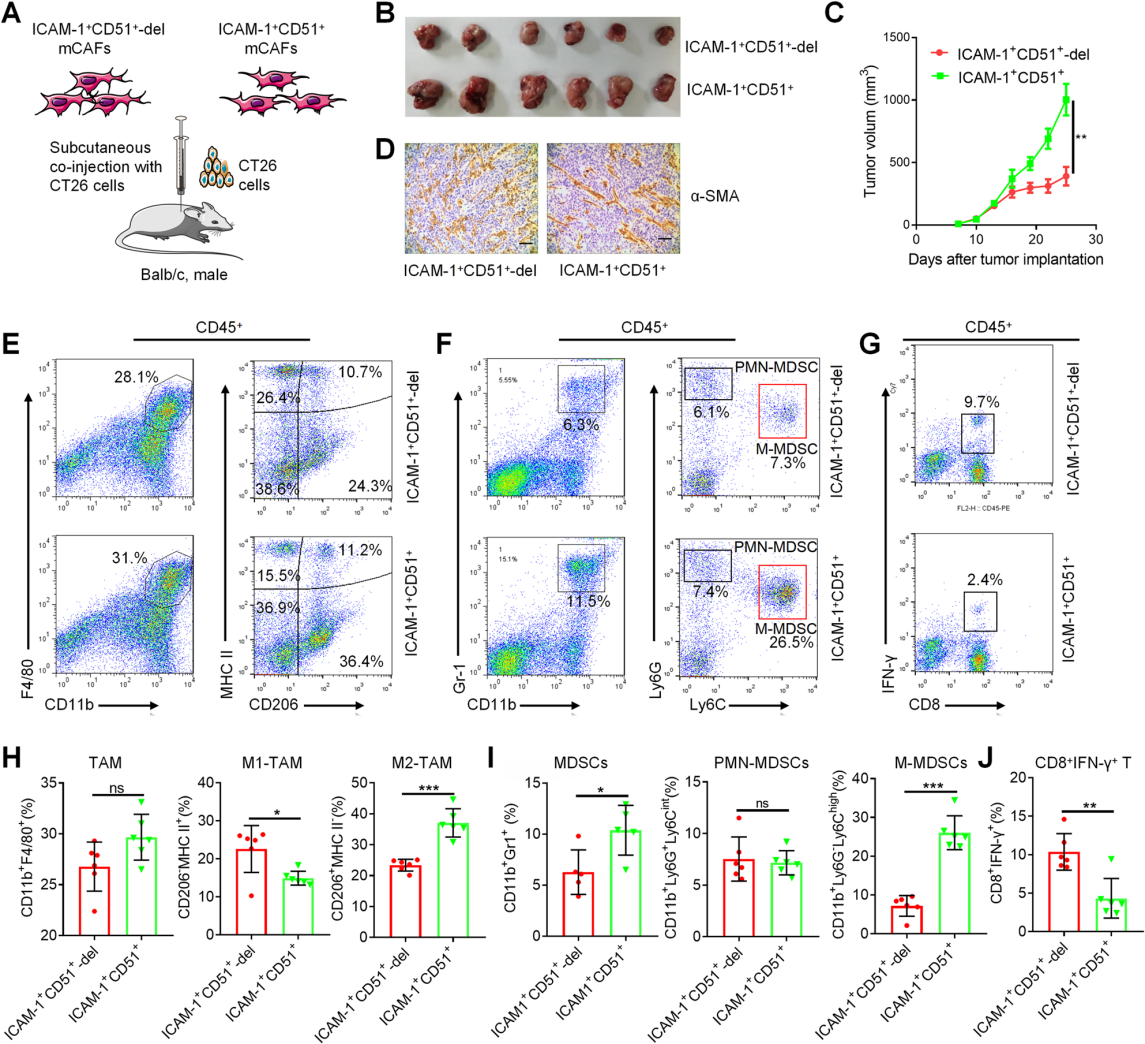
ICAM-1^+^CD51^+^ CAFs increase intratumoral infiltration of TAMs and MDSCs and promote tumor growth. (**A**) Flowchart for the construction of the tumor transplantation model; CT26 cells were co-injected with either ICAM-1^+^CD51^+^ mCAFs or ICAM-1^+^CD51^+^ Del mCAFs into the anterior flank of BALB/c mice (n=6 mice per group, biological replicates). (**B**) Photographs of tumors in the mouse tumor model. Images are representative of n=6 mice per group. (**C**) Statistical analysis of tumor volumes in the mouse tumor model. Data represent mean ± SD from n=6 mice per group (biological replicates). (**D**) Expression and distribution of α-SMA in the tumor tissues of the mouse tumor model (n=6 per group). Scale bar = 100 µm. (**E**) Flow cytometric analysis of the proportions of total TAMs (CD45⁺CD11b⁺F4/80⁺), M1-TAMs (CD45⁺CD11b⁺F4/80⁺CD206⁻MHCII⁺) and M2-TAMs (CD45⁺CD11b⁺F4/80⁺CD206⁺MHCII⁻) within mouse tumor tissues (n=6 per group). (**F**) Flow cytometric analysis of the proportions of total MDSCs (CD45⁺CD11b⁺Gr1⁺), PMN-MDSCs (CD45⁺CD11b⁺Ly6CⁱⁿᵗLy6G⁺), M-MDSCs (CD11b⁺Ly6CʰⁱᵍʰLy6G⁻), within mouse tumor tissues (n=6 per group). (**G**) Flow cytometric analysis of the proportions of CD8⁺IFN-γ⁺ T cells within mouse tumor tissues; (**H**) Statistical analysis of the proportions of total TAMs, M1-TAMs, and M2-TAMs in mouse tumor tissues (n=6 per group). Data represent mean ± SD from n=6 mice per group (two-tailed unpaired Student's t-test). (**I**) Statistical analysis of the proportions of total MDSCs, PMN-MDSCs and M-MDSCs in mouse tumor tissues; Data represent mean ± SD from n=6 mice per group (two-tailed unpaired Student's t-test). (**J**) Statistical analysis of the proportion of CD8⁺IFN-γ⁺ T cells in mouse tumor tissues. Data represent mean ± SD from n=6 mice per group (two-tailed unpaired Student's t-test). ns, not significant; P<0.05, **P<0.01, ***P<0.001

To further investigate the role of ICAM-1^+^CD51^+^ CAFs in the tumor microenvironment (TME), we isolated ICAM-1^+^CD51^+^ mouse CAFs (mCAFs) from mouse-derived mCAFs and co-injected them with CT26 cells to establish a tumor transplantation model (Fig. 4A). We analyzed the proportions of total TAMs, M1-TAMs, M2-TAMs, total MDSCs, polymorphonuclear MDSCs (PMN-MDSCs), monocytic MDSCs (M-MDSCs), and CD8^+^IFNγ^+^ T cells in the tissues. We found that, compared with ICAM-1^+^CD51^+^ Del mCAFs, ICAM-1^+^CD51^+^ mCAFs significantly promoted tumor growth (Fig. 4B-C). The immunohistochemical staining of α-SMA revealed the distribution of the two types of CAFs transplanted in the mouse tumor tissues (Fig. 4D). ICAM-1^+^CD51^+^ mCAFs enhanced the infiltration of M2-type TAMs and reduced the infiltration of M1-type TAMs (Fig. 4E and H). ICAM-1^+^CD51^+^ mCAFs enhanced the infiltration of total MDSCs and M-MDSCs (Fig. 4F and I) in the tumors. Meanwhile, the proportions of CD8^+^IFN-γ^+^ T cells in the tissues of the ICAM-1^+^CD51^+^ mCAFs group were significantly reduced (Fig. 4G and J).

### ICAM-1^**+**^CD51^**+**^CAFs recruit monocytes by secreting MCP-3, CXCL5, and IL-6

**Fig. 5:**
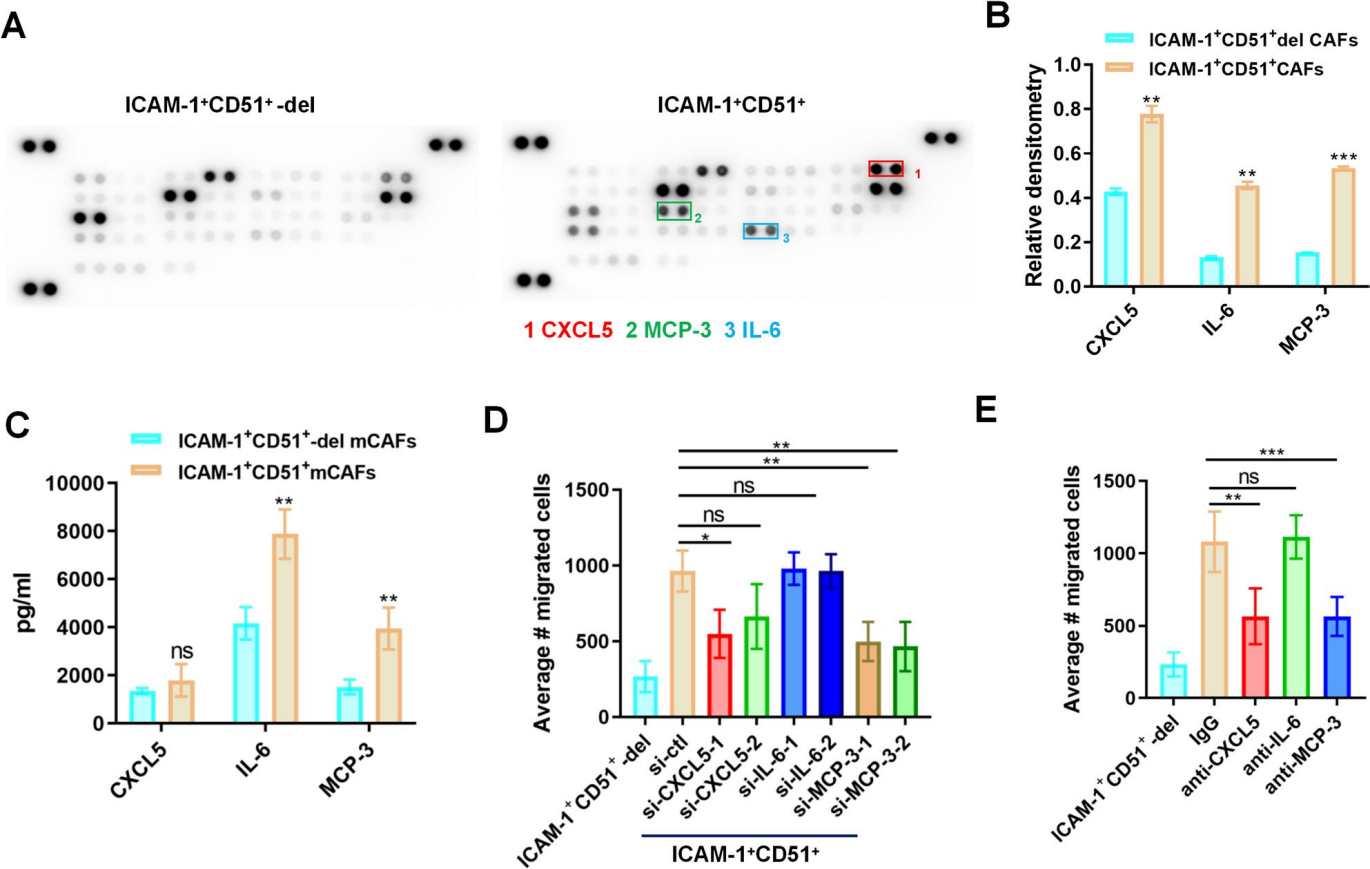
ICAM-1^+^CD51^+^ CAFs recruit monocytes by secreting MCP-3, CXCL5, and IL-6. (A-B) Analysis of cytokine secretion by ICAM-1^+^CD51^+^ CAFs and ICAM-1^+^CD51^+^ Del CAFs using a human cytokine antibody array. The conditioned media were collected from three independent patient-derived CAF isolates per group (*n* = 3 biological replicates). (**A**) Representative array images. (**B**) Quantification of relative cytokine levels. Data represent mean ± SD (two-tailed unpaired Student’s t-test, with Benjamini-Hochberg FDR correction; q-values < 0.05 for all three cytokines). (**C**) Detection of MCP-3, CXCL5, and IL-6 levels in the culture supernatants of mouse-derived ICAM-1^+^CD51^+^ mCAFs and ICAM-1^+^CD51^+^ Del mCAFs (*n* = 4 biological replicates) by ELISA; Data represent mean ± SD of three technical replicates per biological replicate (two-tailed unpaired Student’s t-test). (**D**) Knockdown of MCP-3, CXCL5, and IL-6 expression in ICAM-1^+^CD51^+^ CAFs using small interfering RNAs, followed by analysis of the monocyte-recruiting ability of the treated ICAM-1^+^CD51^+^ CAFs, with flow cytometry used to quantify the number of migrated CD14^+^ monocytes. Data represent mean ± SD from *n* = 4 independent patient-derived CAF isolates (biological replicates), with each condition performed in triplicate (technical replicates). (**E**) Addition of blocking antibodies against MCP-3, CXCL5, IL-6 or isotype control IgG to the culture medium in monocyte chemotaxis assays. The number of migrated CD14⁺ monocytes was quantified by flow cytometry. Data represent mean ± SD from *n* = 4 independent patient-derived CAF isolates (biological replicates), with each condition performed in triplicate (technical replicates). Addition of blocking antibodies against MCP-3, CXCL5, and IL-6 to the culture medium in monocyte chemotaxis assays to analyze the monocyte-recruiting ability of ICAM-1^+^CD51^+^ CAFs (*n* = 3, technical replicates). ns, not significant; *P* < 0.05, **P < 0.01, ***P < 0.001

To further investigate the modes and mechanisms by which ICAM-1^+^CD51^+^ CAFs induce the formation of an immunosuppressive tumor microenvironment, we employed a human cytokine antibody array to screen for differences in cytokine secretion between ICAM-1^+^CD51^+^ CAFs and ICAM-1^+^CD51^+^ Del CAFs in vitro. We found that ICAM-1^+^CD51^+^ CAFs secreted higher levels of MCP-3, CXCL5, and IL-6 (Fig. 5A-B).

It has been reported that MCP-3 and CXCL5 are highly expressed in the tumor microenvironment of various cancers, including CRC, breast cancer, and gastric cancer, where they contribute to TME formation and promote tumor invasion and metastasis [21–23]. CXCL5 primarily exerts a potent chemotactic effect on neutrophils via its receptor CXCR2 [24], and it can also recruit CXCR2⁺ MDSCs into the tumor microenvironment [25, 26]. ELISA analysis of the culture supernatants from isolated mouse-derived ICAM-1^+^CD51^+^ mCAFs and ICAM-1^+^CD51^+^ Del mCAFs revealed that, except for CXCL5, which showed no significant difference, the levels of MCP-3 and IL-6 were significantly elevated (Fig. 5C).

In vitro chemotaxis assays with monocytes demonstrated that antibody blockade of CXCL5 and MCP-3 both attenuated the recruitment of monocytes by ICAM-1^+^CD51^+^ CAFs, whereas blockade of IL-6 had no effect on monocyte recruitment. Consistent results were obtained when CXCL5, MCP-3, and IL-6 expression were knocked down in ICAM-1^+^CD51^+^ CAFs (Fig. 5D-E). We hypothesize that ICAM-1^+^CD51^+^ CAFs may recruit monocytes and induce their differentiation into M2-type TAMs and M-MDSCs by secreting MCP-3, CXCL5, and IL-6, while also recruiting peripheral MDSCs to form an immunosuppressive TME, thereby contributing to CRC immune evasion.

### The OPN-CD51-mediated p38 MAPK pathway may regulate the secretion of MCP-3, CXCL5, and IL-6 by ICAM-1⁺CD51⁺ CAFs

**Fig. 6:**
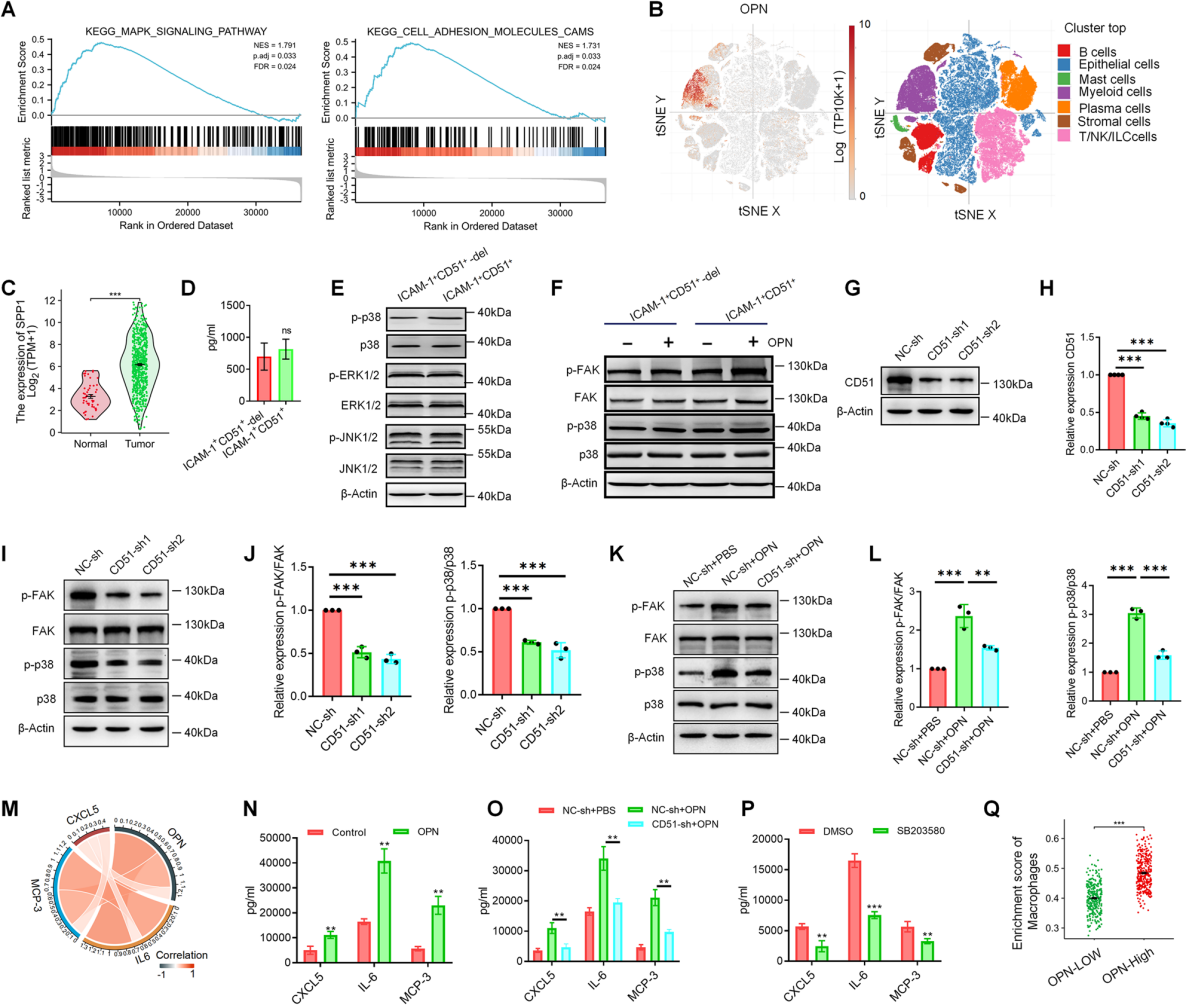
The OPN-CD51-mediated p38 MAPK pathway regulates the secretion of MCP-3, CXCL5 and IL-6. (**A**) GSEA plots showing the signaling pathways enriched in CAFs high recruit CAFs. NES and FDR values are shown. Significance threshold: |NES| > 1, FDR < 0.25. (**B**) Western blot analysis of p38, ERK1/2, JNK1/2 and their phosphorylation levels in ICAM-1⁺CD51⁺ CAFs and ICAM-1⁺CD51⁺Del CAFs (n=3 biological replicates per group). (**C**) Expression analysis of OPN in single-cell RNA-seq data from colorectal cancer. UMAP plot shows OPN expression across cell clusters. Data are representative of the dataset. (**D**) Analysis of SPP1 expression in CRC (n=698) versus normal tissues (n=51) using the TCGA database. Mann-whitney U test was used. (**E**) ELISA measurement of OPN levels in the conditioned medium from cultured ICAM-1⁺CD51⁺ CAFs and ICAM-1⁺CD51⁺Del CAFs. Data represent mean ± SD of three technical replicates（two-tailed unpaired Student's t-test. (**F**) Western blot analysis of p38, FAK, p-p38, and p-FAK levels in ICAM-1⁺CD51⁺ CAFs and ICAM-1⁺CD51⁺Del CAFs following OPN stimulation (n=3 biological replicates per group). (**G**) Western blot analysis of CD51 in ICAM-1⁺CD51⁺ CAFs after siRNA-mediated CD51 knockdown (n=3 biological replicates per group). (**H**) Western blot analysis of p38, FAK, p-p38, and p-FAK protein levels in ICAM-1⁺CD51⁺ CAFs after siRNA-mediated CD51 knockdown (n=3 biological replicates per group). (**I-J**) Densitometric quantification of protein bands was performed and the CD51 protein levels in figure 6H were normalized to the loading control β-actin. (**K**) ICAM-1⁺CD51⁺ CAFs and ICAM-1⁺CD51⁺ CAFs after siRNA-mediated CD51 knockdown were treated with OPN. Western blot analysis of p38, FAK, p-p38, and p-FAK protein levels (n=3 biological replicates per group). (**L**) Densitometric quantification of protein bands was performed and the CD51 protein levels in figure 6K were normalized to the loading control β-actin. (**M**) Correlation analysis of OPN, CXCL5, MCP-3 and IL-6 expression in CRC using TCGA data (n=698). The outermost labels in a chord diagram denote the variables. Chord width reflects the correlation strength between two variables, and the node width equals the total width of all chords attached to it. (**N**) ELISA measurement of MCP-3, CXCL5, and IL-6 levels in the conditioned medium from ICAM-1⁺CD51⁺ CAFs following OPN stimulation. Values represent mean ± SD of three technical replicates (two-tailed unpaired Student's t test). (**O**) ICAM-1⁺CD51⁺ CAFs and ICAM-1⁺CD51⁺ CAFs after siRNA-mediated CD51 knockdown were treated with OPN. ELISA measurement of MCP-3, CXCL5, and IL-6 levels in the conditioned medium from indicated CAFs following OPN stimulation. Values represent mean ± SD of three technical replicates (two-tailed unpaired Student's t test). (**P**) ICAM-1⁺CD51⁺ CAFs were pretreated with the p38 inhibitor SB203580 (10 µM) or DMSO prior to OPN stimulation. ELISA measurement of MCP-3, CXCL5, and IL-6 levels in the conditioned medium from indicated CAFs following OPN stimulation.Values represent mean ± SD of three technical replicates (two-tailed unpaired Student's t test). (**Q**) Comparison of macrophage infiltration scores between CRC tissues with high and low OPN expression. ns, not significant; **P<0.01, ***P<0.001

Bioinformatics analysis revealed that CAFs with high recruitment capacity (CAFs High recruit) exhibited enrichment in the activation of multiple signaling pathways, including notably altered pathways such as cell adhesion molecules and the MAPK signaling pathway (Fig. 6 A). Upon examining the MAPK signaling pathway in ICAM-1⁺CD51⁺ CAFs and ICAM-1⁺CD51⁺Del CAFs, we found increased levels of p38 phosphorylation in ICAM-1⁺CD51⁺ CAFs, while the phosphorylation levels of ERK and JNK showed no significant change (Fig. 6B and Supplementary Fig. 4 A). Activation of the MAPK pathway regulates nuclear translocation of phosphorylated transcription factors (e.g., AP-1, c-Myc, CREB), thereby promoting cytokine expression [27, 28]. The promoters of MCP-3, CXCL5, and IL-6 contain binding sites for these factors. Additionally, p38 MAPK regulates MCP-3 secretion in dermal fibroblasts [29]. Therefore, the p38 MAPK pathway may also regulate the secretion of these three chemokines in ICAM-1⁺CD51⁺ CAFs.

CD51, as an important member of the integrin family, serving as a recognition receptor for osteopontin (OPN), fibronectin, and vitronectin [30, 31]. OPN is a glycoprotein encoded by the SPP1 gene and is considered a cytokine involved in various physiological and pathological processes. OPN is highly expressed in CRC, and elevated serum/plasma OPN levels are closely associated with a high incidence of metastasis and poor prognosis [32]. Single-cell RNA sequencing data from CRC tissues indicated that OPN are mainly expressed within a subset of myeloid cells and stromal cells (Fig. 6C). Using the TCGA database, we confirmed the high expression of OPN in CRC tissues (Fig. 6D). We detected a certain amount of OPN in the conditioned medium of ICAM-1⁺CD51⁺ CAFs cultured (Fig. 6E). Further experiments demonstrated that OPN induces the activation of the p38 MAPK pathway and the phosphorylation of FAK, a downstream molecule of CD51, in ICAM-1⁺CD51⁺ CAFs. In contrast, OPN had no effect on the p38 MAPK pathway or FAK phosphorylation in ICAM-1⁺CD51⁺Del CAFs (Fig. 6 F and Supplementary Fig. 4B).

Next, we used siRNA targeting CD51 to knock down CD51 expression in ICAM-1⁺CD51⁺ CAFs (Fig. 6G). As shown in new Fig. 6H-J, CD51 knockdown significantly reduced the basal phosphorylation levels of FAK and p38 in ICAM-1⁺CD51⁺ CAFs. CD51 knockdown significantly reduced OPN-induced p38 and FAK phosphorylation (Fig 6K-L).These results suggest that OPN may activate the FAK and downstream p38 MAPK pathway by binding to the CD51 receptor on the surface of ICAM-1⁺CD51⁺ CAFs.

Furthermore, the expression level of SPP1 in CRC was significantly positively correlated with the expression levels of CXCL5, MCP-3, and IL-6 (Fig. 6M). Additionally, OPN was able to stimulate the secretion of MCP-3, CXCL5, and IL-6 by ICAM-1⁺CD51⁺ CAFs (Fig. 6 N). CD51 knockdown also decreased the level of MCP-3, CXCL5, and IL-6 in the culture supernatants of ICAM-1⁺CD51⁺ CAFs (Fig 6O). Pharmacological inhibition of p38 MAPK by p38 inhibitor SB203580 markedly suppressed the secretion of MCP-3, CXCL5, and IL-6 (Fig. 6P). These data establish a direct causal link between the OPN-CD51-FAK/p38 axis and chemokine secretion. CRC tumor tissues with high OPN (coding by SPP1) expression showed a significantly higher macrophage infiltration score compared to those with low OPN expression (Fig. 6Q), and OPN expression was positively correlated with macrophage enrichment (*r* = 0.788, *p* < 0.001, and Supplementary Figure. 5). Taken together, these results suggest a potential mechanism whereby OPN, acting through CD51, activates the FAK/p38 MAPK pathway, thereby promoting the secretion of MCP-3, CXCL5, and IL-6. This cascade may thereby promote monocyte recruitment and acquire M2-like TAM/M-MDSC-associated phenotype, ultimately facilitating immune evasion in CRC.

## Discussion 

ICAM-1, an immunoglobulin‑like transmembrane glycoprotein, mediates cell‑cell adhesion and ECM interactions. In tumor progression, it drives hepatic metastasis by facilitating tumor cell adhesion, pro‑metastatic signaling, immune cell recruitment, and angiogenesis [33, 34]. Elevated serum levels of ICAM-1 in CRC patients correlate with poor survival outcomes [35]. CD51 (integrin αv ) dimerizes with β subunits to bind fibronectin, vitronectin, and OPN. High CD51 expression in CRC is linked to advanced TNM stage, metastasis, and chemoresistance [36, 37]. However, the roles of ICAM-1 and CD51 in CAFs remain poorly understood.

We found that ICAM-1 and CD51 are differentially expressed between CAFs with high versus low monocyte recruitment capacity. Both proteins localize to the tumor stroma and co-localize with α-SMA⁺ fibroblasts. The high‑recruit group contained significantly more ICAM-1⁺CD51⁺ CAFs, which showed superior monocyte recruitment, drived monocytes to acquire M2-like TAM/M-MDSC-associated phenotype, and promoted TAM/MDSC infiltration and tumor growth in mice. Thus, ICAM‑1 and CD51 serve as effective surface markers for distinguishing CAF subpopulations based on monocyte chemotactic capacity.

Based on our data, the ICAM-1⁺CD51⁺ CAF subset exhibits mixed features and cannot be simply assigned to classical iCAF or myCAF categories. Functionally, it resembles iCAFs by promoting immunosuppression via IL-6 and chemokines. However, PDGFRA (an iCAF marker) showed no differential expression between high- and low-recruit groups, while α-SMA (a myCAF hallmark) was similarly expressed in both groups and co-localized with ICAM-1 and CD51. Flow cytometry confirmed that both sorted subsets are α-SMA⁺ with comparable mean fluorescence intensity, and FAP was positive in both. Sorted ICAM-1⁺CD51⁺ CAFs retained > 95% double-positive expression after prolonged culture and cryopreservation-reculture, supporting relative stability in vitro. Nonetheless, we acknowledge that CAF phenotypes are plastic and influenced by microenvironmental cues; our “mixed/intermediate” description therefore denotes functional divergence from classical classifications rather than a transient state. We thus cautiously regard ICAM-1⁺CD51⁺ CAFs as a marker-defined, functionally enriched population with mixed features.

We acknowledge that our in vitro experiments used conventional 2D or Transwell systems lacking an extracellular matrix (ECM), which does not fully recapitulate the complex 3D architecture of the tumor stroma. Future validation using 3D organoid or ECM-embedded models would be valuable. Nevertheless, our in vivo mouse models provide complementary evidence supporting the functional role of ICAM-1⁺CD51⁺ CAFs within an intact TME. We analyzed overall survival in our cohort stratified by ICAM-1⁺CD51⁺ CAF abundance and found no significant difference. This lack of significance likely reflects limited statistical power due to small sample size, short follow-up (median 36 months), and heterogeneous adjuvant regimens. To address these limitations, we plan to validate the clinical significance of this CAF subset in an expanded cohort of patients with a five year follow-up, enabling robust survival analyses.

Our screening with human cytokine antibody arrays revealed that ICAM-1⁺CD51⁺ CAFs secrete elevated levels of MCP-3, CXCL5, and IL-6. MCP-3 and CXCL5 are highly expressed in the tumor microenvironment of various cancers [21–23]. CXCL5 exerts a potent chemotactic effect on neutrophils mainly through its receptor CXCR2 [24], and it can recruit CXCR2⁺ MDSCs into the tumor microenvironment [25, 26]. Given that CXCR2 is expressed on some monocytes, we hypothesized that CAF‑derived CXCL5 recruits monocytes via CXCR2 to promote TAM accumulation. Indeed, blocking or knocking down CXCL5 or MCP‑3, but not IL‑6, reduced monocyte recruitment by ICAM‑1⁺CD51⁺ CAFs in vitro. Although IL-6 blockade did not affect monocyte recruitment, IL-6 from ICAM-1⁺CD51⁺ CAFs likely drives monocyte differentiation and immunosuppression rather than chemotaxis. IL-6 directs monocyte differentiation toward macrophages and TAM-like cells [38], promotes M2 polarization, and upregulates PD-L1 via STAT3 signaling [39, 40]. Thus, CAF-derived IL-6 may primarily contributes to differentiation and immune suppression, not recruitment.

MCP-3 upregulation in CRC patients correlates with poor prognosis and liver metastasis [41, 42]. As a chemokine, MCP-3 can recruit monocytes, dendritic cells, eosinophils, basophils, and other immune cells by binding to its receptors CCR1, CCR2, and CCR3 [22]. Moreover, CCR2, one of the receptors for MCP-3, is also expressed on MDSCs [22]. Thus, MCP‑3 may recruit peripheral CCR2⁺ MDSCs via CCR2 to promote immunosuppression, and ICAM‑1⁺CD51⁺ CAFs likely recruit monocytes and MDSCs and drive monocyte differentiation through MCP‑3, CXCL5, and IL‑6 secretion. We acknowledge that MCP-3, CXCL5, and IL-6 can also be produced by other TME cells (e.g., tumor cells, myeloid cells), suggesting functional redundancy. Although ICAM‑1⁺CD51⁺ CAFs secreted higher levels of these cytokines than ICAM‑1⁺CD51⁺ del CAFs (Fig. 5A-B), we did not directly compare them with other cell types, and other chemokines (e.g., CCL2, CXCL1) may play redundant roles. Future in vivo studies using conditional knockout or neutralizing antibodies are needed to determine the non-redundant contribution of this axis.

Mechanistically, we demonstrated that the OPN-CD51 signaling axis phosphorylates and activates the FAK/p38 MAPK pathway, driving the secretion of MCP-3, CXCL5, and IL-6 in ICAM 1⁺CD51⁺ CAFs in vitro. It has been reported that activation of the MAPK pathway can regulate the nuclear translocation of various phosphorylated transcription factors, including AP-1, c-Myc, and CREB, thereby promoting cytokine expression [27, 28]. The promoter regions of MCP-3, CXCL5, and IL-6 contain binding sites for MAPK-regulated transcription factors. Studies have shown that in dermal fibroblasts, p38 MAPK is involved in regulating the secretion of chemokines such as MCP-3 [29]. Given the well-documented role of the p38 MAPK pathway in regulating ICAM-1 expression [43, 44], we hypothesize that activation of the p38 MAPK pathway in ICAM-1⁺CD51⁺ CAFs contributes to maintaining their functions.

CD51 forms heterodimers with β subunits and serves as a receptor for OPN, fibronectin, and vitronectin [30, 31]. OPN is highly expressed in CRC and associated with poor prognosis [32]. In this study, we detected a certain amount of OPN in the conditioned medium of ICAM-1⁺CD51⁺ CAFs. Vitronectin binding to CD51 induces p38 MAPK activation and enhances breast cancer invasion and metastasis [45]. Moreover, OPN can regulate FAK, ERK1/2, p38, and NF-κB signaling pathways, thereby participating in immune responses and tumor progression. Our experiments further demonstrated that OPN activated the p38 MAPK/FAK pathway in ICAM-1⁺CD51⁺ CAFs and enhanced MCP-3, CXCL5, and IL-6 secretion, suggesting its role in maintaining their functional phenotype. However, this study did not delve deeper into the functional contribution of OPN to the activation. A potential limitation of targeting CD51 lies in its capacity to pair with several subunits and its presence on diverse cell populations, such as endothelial cells and osteoclasts. Consequently, therapeutic strategies directed against CD51 carry a risk of off-target effects and associated toxicities. Additionally, it is not yet known which β-integrin subunit partner with CD51 to execute OPN-triggered activation of the FAK/p38 MAPK pathway within ICAM-1⁺CD51⁺ CAFs.

**Fig. 7:**
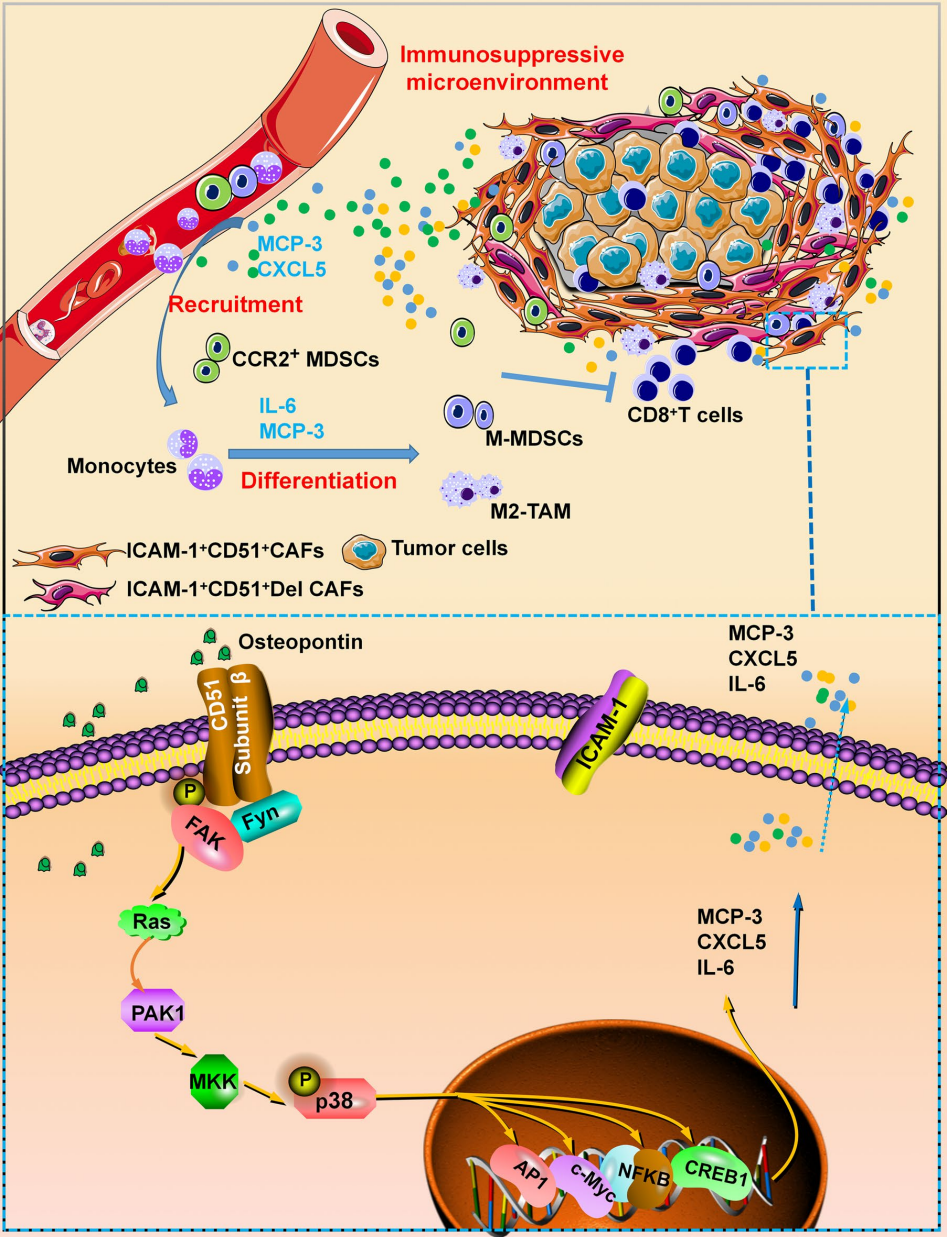
Schematic illustration of how ICAM-1⁺CD51⁺ CAFs mediate immune evasion in CRC regulating the immunosuppressive microenvironment. Osteopontin binds to CD51 integrin on the surface of ICAM-1⁺CD51⁺ CAFs, activating the FAK/p38 MAPK signaling pathway within these CAFs. Activated CAFs then secrete elevated levels of MCP-3, CXCL5, and IL-6. MCP-3 and CXCL5 act as chemoattractants to recruit circulating monocytes into the tumor microenvironment, while IL-6 drives the differentiation of recruited monocytes into immunosuppressive M2-like TAMs and M-MDSCs. These myeloid cells in turn suppress CD8⁺IFN-γ⁺ T-cell function, promote tumor growth, and facilitate immune evasion

This study identifies a functionally enriched, marker-defined CAF subset co-expressing ICAM-1 and CD51 that, via OPN/CD51 mediated FAK/p38 activation, secretes MCP-3, CXCL5, and IL-6 to recruit and drives monocytes to acquire M2-like phenotype and suppressive features characteristic of M2-TAMs and M-MDSCs, thereby fostering immunosuppression and immune evasion.(Fig. 7). This observation does not preclude the existence of other immunoregulatory CAF subsets previously described, but rather adds a marker-defined axis to the current understanding. From a translational perspective, ICAM-1⁺CD51⁺ CAFs may be enriched in aggressive, advanced-stage CRC, as high CD51 expression correlates with residual tumor and lymphatic invasion. Their strong association with TAM infiltration and CD8⁺ T cell exhaustion markers suggests they contribute to immune-cold tumors and potential resistance to immunotherapy. Thus, targeting this CAF subset could synergize with immunotherapies by alleviating myeloid suppression.

This work highlights the potential of targeting ICAM-1⁺CD51⁺ CAFs as a therapeutic strategy for CRC. Several potential therapeutic strategies can be considered based on our findings: directly targeting ICAM-1⁺CD51⁺ CAFs with antibody-drug conjugates (ADCs) against ICAM-1 or CD51; blocking the OPN-CD51 axis with neutralizing antibodies or small-molecule integrin inhibitors; inhibiting downstream FAK/p38 MAPK signaling with clinically available agents. By elucidating the molecular mechanisms through which this CAF subpopulation contributes to immunosuppression, our findings provide novel insights and potential targets for CRC treatment strategies aimed at modulating the tumor microenvironment.

## Supplementary Information

Below is the link to the electronic supplementary material.

Supplementary Material 1

Supplementary Material 2

## Data Availability

The datasets used and/or analyzed during the current study are available from the corresponding author on reasonable request.

## Abbreviations

CAFs: Cancer-associated fibroblasts
CRC: Colorectal cancer
OPN: Osteopontin
TAMs: Tumor-associated macrophages
MDSCs: Myeloid-derived suppressor cells
MCP-3: Monocyte chemoattractant protein-3
CXCL5: C-X-C motif chemokine ligand 5
IL-6: Interleukin-6
M-MDSCs: Monocytic myeloid-derived suppressor cells
M2-TAMs: M2-polarized tumor-associated macrophages
TME: Tumor microenvironment
PMN-MDSC: Polymorphonuclear myeloid-derived suppressor cells
TCGA: The cancer genome atlas
GTEx: Genotype-tissue expression
GEO: Gene expression omnibus
GSEA: Gene set enrichment analysis
BSA: Bovine serum albumin
HRP: Horseradish peroxidase
