# Supplementary material for: ICAM-1⁺CD51⁺ CAFs drive immunosuppression in colorectal cancer via OPN-triggered chemokine secretion
Source: J Transl Med. 2026 Jul 17;24:957. doi: 10.1186/s12967-026-08642-9 (PMC13397660; doi:10.1186/s12967-026-08642-9)
Supplement: Supplementary file 1 — Supplementary Material 1 [file 12967_2026_8642_MOESM1_ESM.doc]

**ICAM-1⁺CD51⁺ CAFs Drive Immunosuppression in Colorectal Cancer via OPN-Triggered Chemokine Secretion**

Jia Liu^1#^, Senrui Xue^2#^, Yixin Xu^3,4#^, Sicheng Wu^2^, Wenyu Zhao^5^, Xinmiao Li^5^, Nan Hu^5^, Jinmin Sun^5*^, Jing Ren^2*^

1 Department of Pathology, The Affiliated Hospital of Xuzhou Medical University， Xuzhou Medical University, Xuzhou, 221004, China.

2. Jiangsu Key Laboratory of Brain Disease Bioinformation, Research Center for Biochemistry and Molecular Biology, Xuzhou Medical University, Xuzhou, 221004, China.

3 Department of General Surgery, The Affiliated Hospital of Xuzhou Medical University, Xuzhou, Jiangsu, China

4. Institute of Digestive Diseases, Xuzhou Medical University, Xuzhou, Jiangsu, China

5 Laboratory of Clinical and Experimental Pathology, Department of Pathology, Xuzhou Medical University, Xuzhou, Jiangsu, China

* Corresponding authors.

E-mail addresses: [renjing@xzhmu.edu.cn (J](mailto:renjing@xzhmu.edu.cn (J). Ren), and sunjinmin09@xzhmu.edu.cn (J. Sun)

# Equal Contribution and first authorship: These authors contributed equally to this work and share first authorship.

**Supplementary Figures**

**
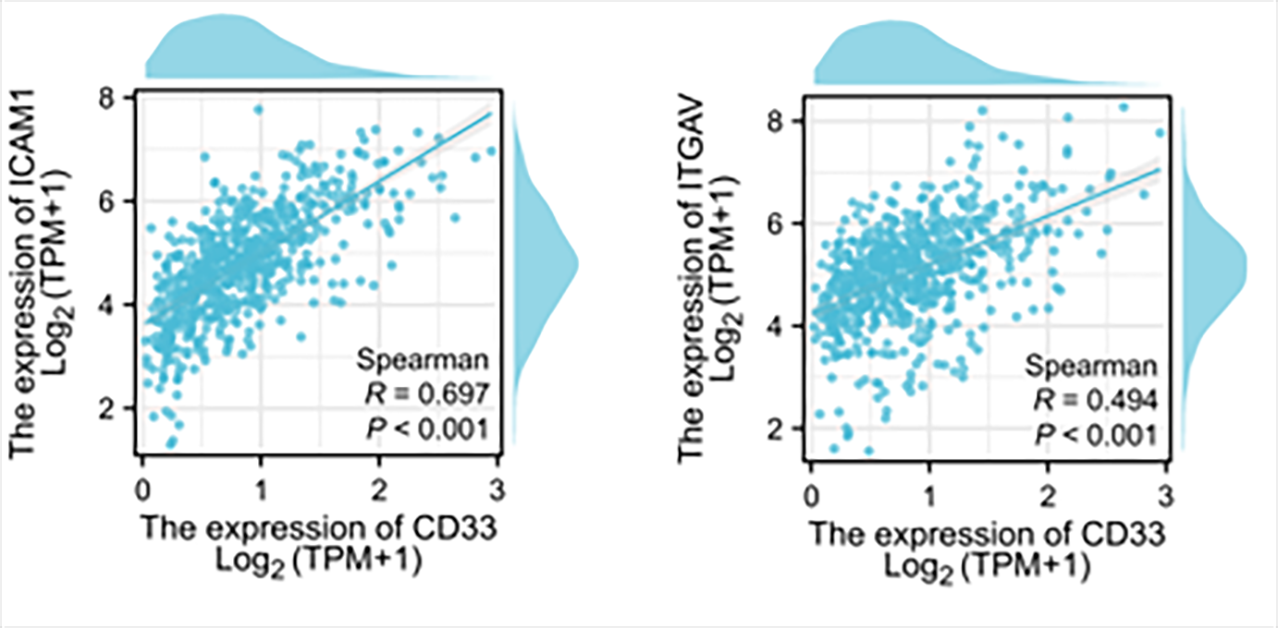
**

**Figure S1.Correlation analysis of ICAM-1 and CD51 (ITGAV) expression with CD33 levels in CRC samples from the TCGA database.** Scatter plots showing the correlation between ICAM-1, ITGAV, and CD33 transcript levels in colorectal cancer patient samples from the TCGA database.


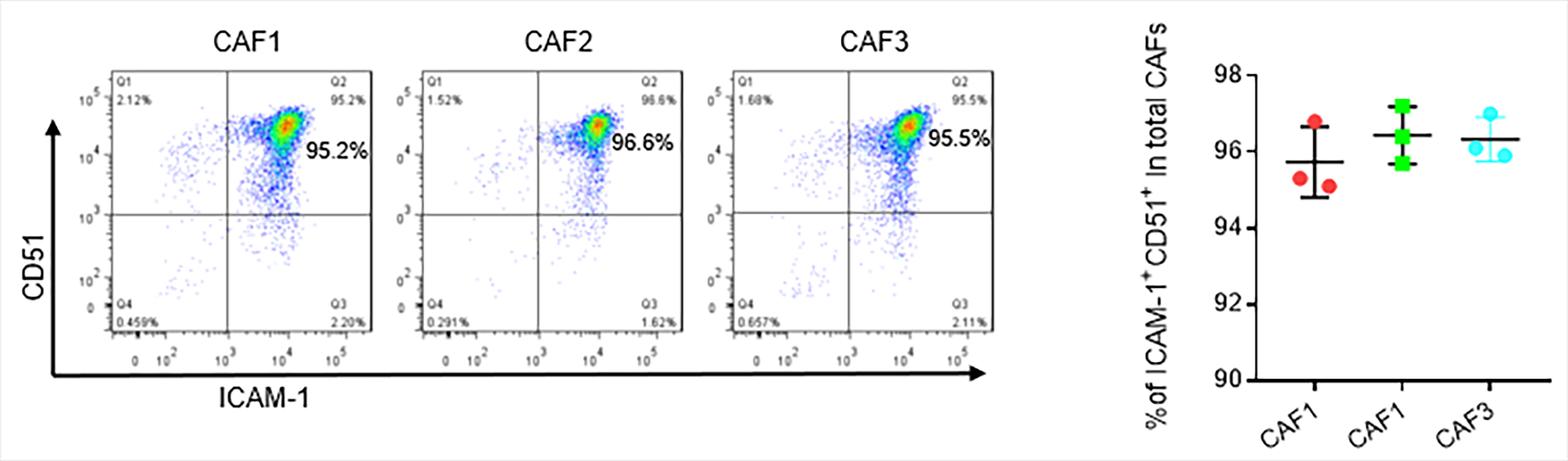
**Figure S2.** **Frequency of ICAM-1^+^CD51^+^ CAFs derived from distinct pathological sources.** Left: Representative flow cytometry plots showing the percentage of double-positive cells. Right: Summary bar graph of the percentage of ICAM-1⁺CD51⁺ CAFs.

**
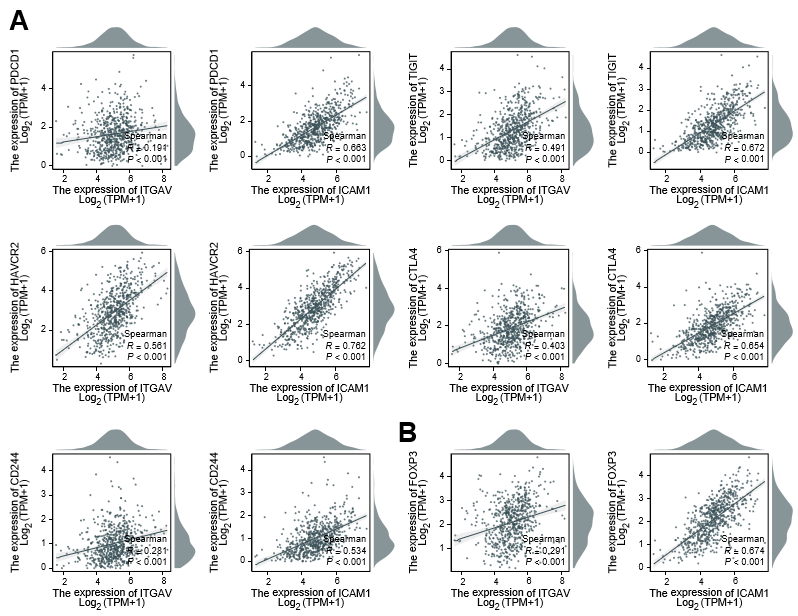
**

**Figure S3.** **Correlation of ICAM-1 and ITGAV expression with CD8⁺ T-cell exhaustion markers and Treg markers in CRC samples from the TCGA database.** (A) Sscatter plots showing the correlations between ICAM-1/ITGAV expression and the indicated immune markers, including CD8⁺ T-cell exhaustion markers (PD-1/PDCD1, TIGIT, TIM-3/HAVCR2, CTLA4, 2B4/CD244). (B) Sscatter plots showing the correlations between ICAM-1/ITGAV expression and the Treg marker FOXP3.

**
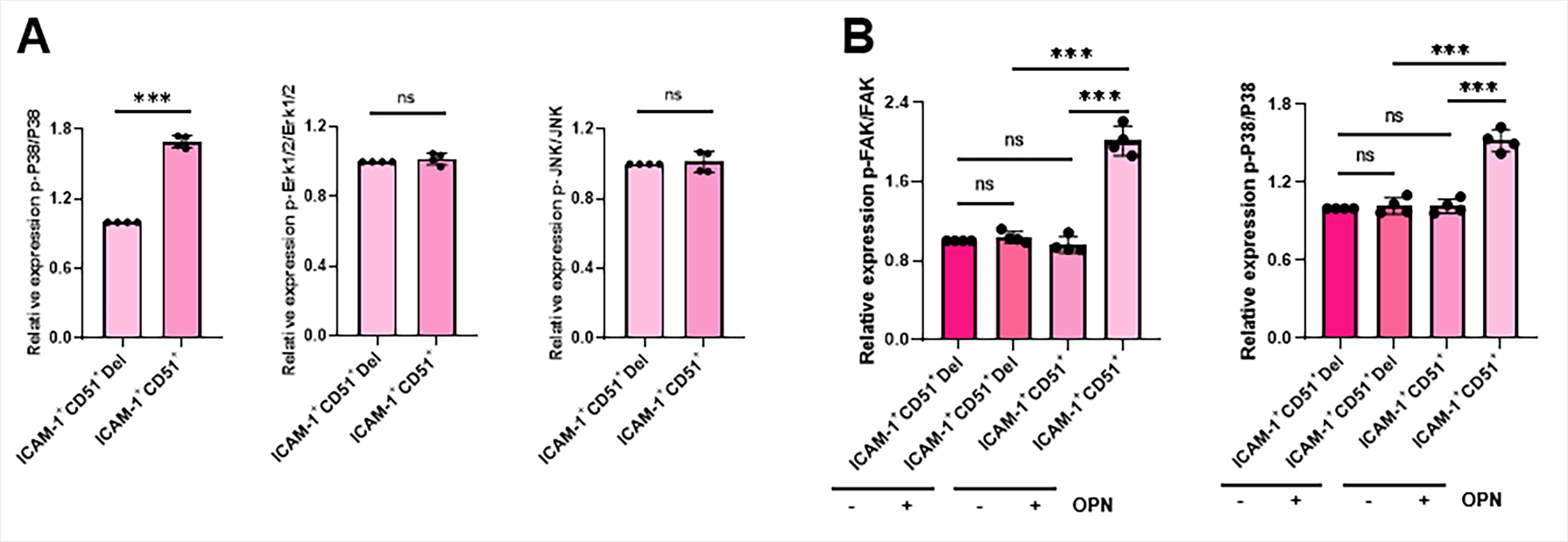
**

**Figure S4. Quantitative statistical analysis of Western blot results shown in Figure 6.** Densitometric quantification of protein bands was performed and the target protein levels were normalized to the loading control β-actin. (A) Quantitative analysis of protein expression in Figure 6B. (B) Quantitative analysis of protein expression in Figure 6F. Data are presented as mean ± SEM from three independent experiments (n = 3). ***p < 0.001, ns: not significant.


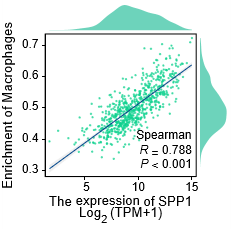


**Figure S5. Correlation analysis between OPN (SPP1) expression levels and macrophage infiltration scores in CRC using the TCGA database.**

**Supplementary Table 1**

| Antibody name | Asage and dilution ratio | Company | Clone Number |
| --- | --- | --- | --- |
| p38 | WB 1:1200, IHC 1:150 | Proteintech | Polyclonal |
| ERK1/2 | WB 1:1000, IHC 1:200 | Proteintech | 4G8A11 |
| JNK1/2 | WB 1:2000 | Proteintech | Polyclonal |
| FAK | WB 1:1000 | Abclonal | ARC0171 |
| P-p38 | WB 1:1000, IHC 1:100 | Santa Cruz | D-8 |
| P-ERK1/2 | WB 1:1000 | Santa Cruz | E-4 |
| P-JNK1/2 | WB 1:1000 | Santa Cruzh | G-7 |
| P-FAK | WB 1:1000, IHC 1:150 | Santa Cruz | 2D11 |
| β-actin | WB 1:20000 | Proteintech | 2D4H5 |
| ICAM-1 | IHC 1:5000, IF 1:300 | Proteintech | Polyclonal |
| CD51 | IHC 1:1500, IF 1:500 | Proteintech | 242376C5 |
| α-SMA  Human CD51  Human ICAM-1  Human α-SMA  Human CD45  Human EPCAM  Human CD31  Human CD11b  Human CD14  Human CD33  Human CD206  Human HLA-DR  Mouse CD51  Mouse ICAM-1  Mouse CD45  Mouse CD11b  Mouse CD206  Mouse F4/80  Mouse MHC II  Mouse Gr-1  Mouse Ly6G  Mouse Ly6C  Mouse CD8  Mouse IFN-γ  Alexa Fluor 488-conjugated anti-rabbit  Alexa Fluor 594-conjugated anti-mouse  Alexa Fluor Plus 647-conjugated anti-goat | IF 1:10000, IF 1:2000  FC 5 µl/10^6^ cells  FC 5 µl/10^6^ cells  FC 5 µl/10^6^ cells  FC 5 µl/10^6^ cells  FC 5 µl/10^6^ cells  FC 5 µl/10^6^ cells  FC 5 µl/10^6^ cells  FC 5 µl/10^6^ cells  FC 5 µl/10^6^ cells  FC 5 µl/10^6^ cells  FC 5 µl/10^6^ cells  FC 5 µl/10^6^ cells  FC 5 µl/10^6^ cells  FC 5 µl/10^6^ cells  FC 5 µl/10^6^ cells  FC 5 µl/10^6^ cells  FC 5 µl/10^6^ cells  FC 5 µl/10^6^ cells  FC 5 µl/10^6^ cells  FC 5 µl/10^6^ cells  FC 5 µl/10^6^ cells  FC 5 µl/10^6^ cells  FC 5 µl/10^6^ cells  IF 1:1000  IF 1:800  IF 1:800 | Proteintech  Biolegend  Biolegend  Biolegend  Biolegend  Biolegend  Biolegend  Biolegend  Biolegend  Biolegend  Biolegend  Biolegend  Biolegend  Biolegend  Biolegend  Biolegend  Biolegend  Biolegend  Biolegend  Biolegend  Biolegend  Biolegend  Biolegend  Biolegend  Invitrogen  Invitrogen  Invitrogen | Polyclonal  NKI-M9  HA58  1A4  HI30  9C4  WM59  ICRF44  HCD14  WM53  15-2  L243  RMV-7  YN1/1.7.4  30-F11  M1/70  C068C2  BM8  M5/114.15.2  RB6-8C5  1A8  HK1.4  53-6.7  XMG1.2  Polyclonal  Polyclonal  Polyclonal |
