# Supplementary material for: ICAM-1⁺CD51⁺ CAFs drive immunosuppression in colorectal cancer via OPN-triggered chemokine secretion
Source: J Transl Med. 2026 Jul 17;24:957. doi: 10.1186/s12967-026-08642-9 (PMC13397660; doi:10.1186/s12967-026-08642-9)
Supplement: Supplementary file 2 — Supplementary Material 2 [file 12967_2026_8642_MOESM2_ESM.pptx]

## Slide 1
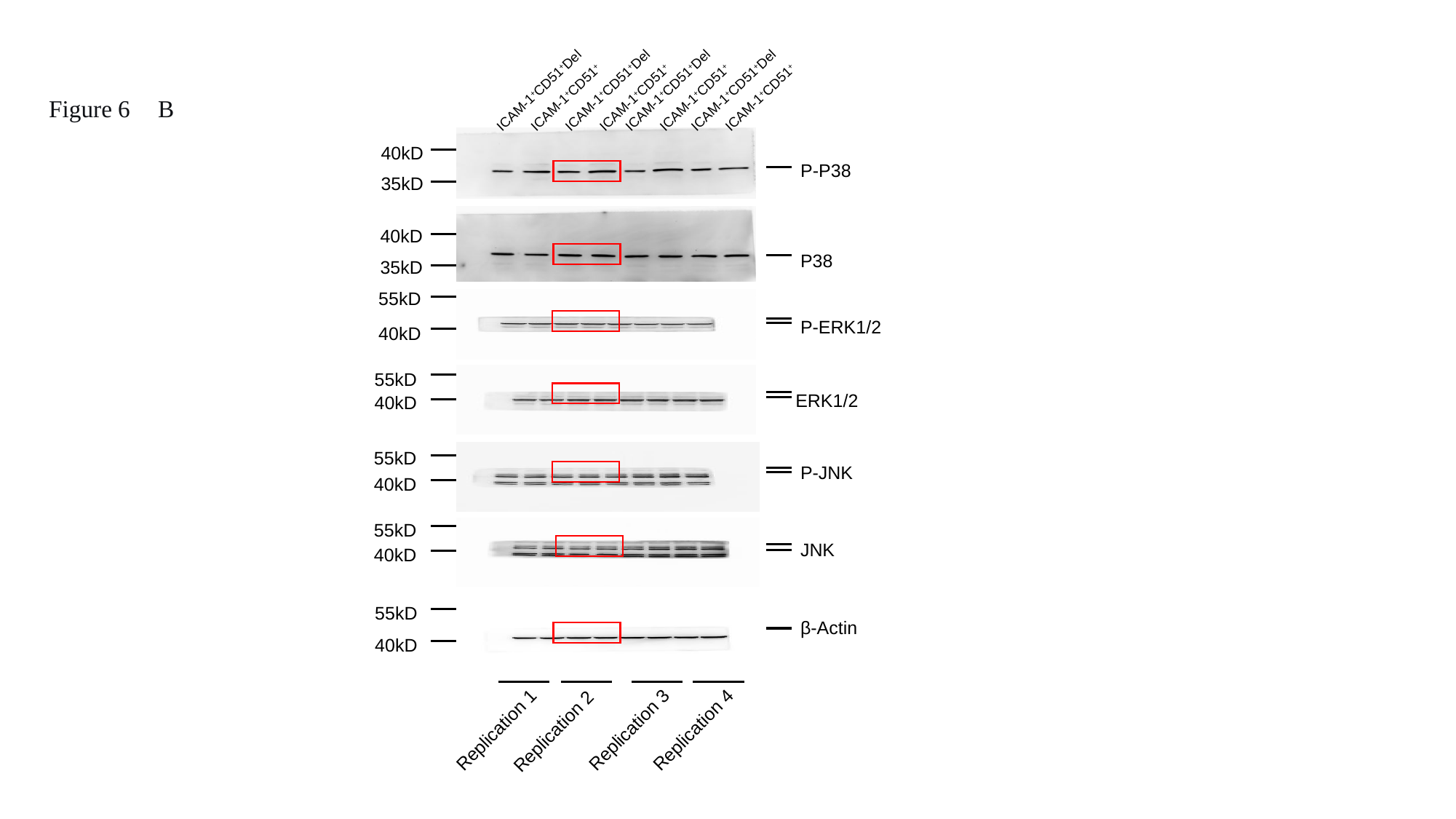

ICAM-1+CD51+Del
ICAM-1+CD51+
ICAM-1+CD51+Del
ICAM-1+CD51+
ICAM-1+CD51+Del
ICAM-1+CD51+
ICAM-1+CD51+Del
ICAM-1+CD51+
Figure 6	B
40kD
P-P38
35kD
40kD
P38
35kD
55kD
P-ERK1/2
40kD
55kD
ERK1/2
40kD
55kD
P-JNK
40kD
55kD
JNK
40kD
55kD
β-Actin
40kD
Replication 1
Replication 3
Replication 4
Replication 2

## Slide 2
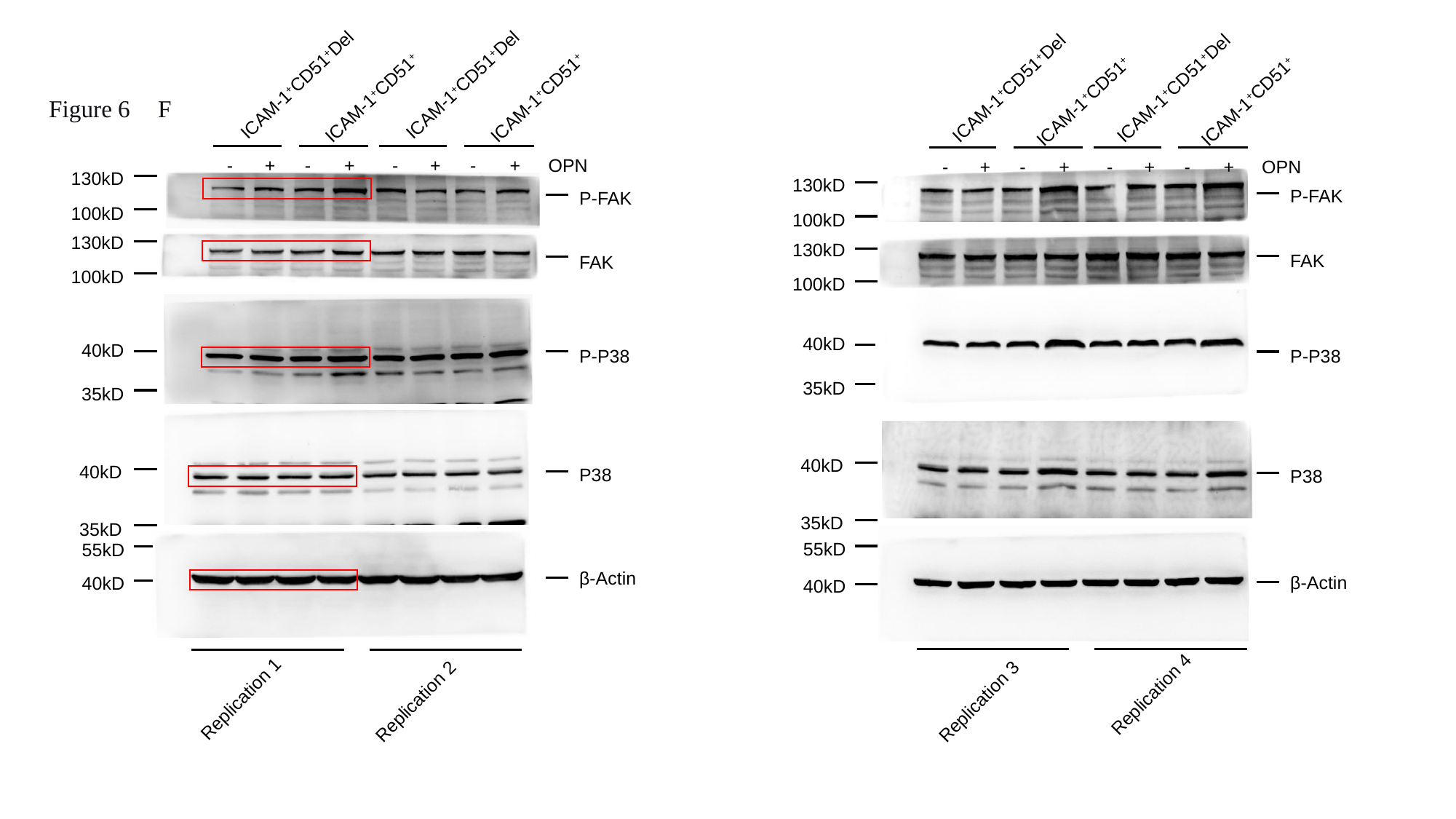

ICAM-1+CD51+Del
ICAM-1+CD51+Del
ICAM-1+CD51+
ICAM-1+CD51+
ICAM-1+CD51+Del
ICAM-1+CD51+Del
ICAM-1+CD51+
ICAM-1+CD51+
-
+
-
+
-
+
-
+
OPN
130kD
P-FAK
100kD
130kD
FAK
100kD
40kD
P-P38
35kD
40kD
P38
35kD
55kD
β-Actin
40kD
Figure 6	F
-
+
-
+
-
+
-
+
OPN
130kD
P-FAK
100kD
130kD
FAK
100kD
40kD
P-P38
35kD
40kD
P38
35kD
55kD
β-Actin
40kD
Replication 4
Replication 1
Replication 2
Replication 3

## Slide 3
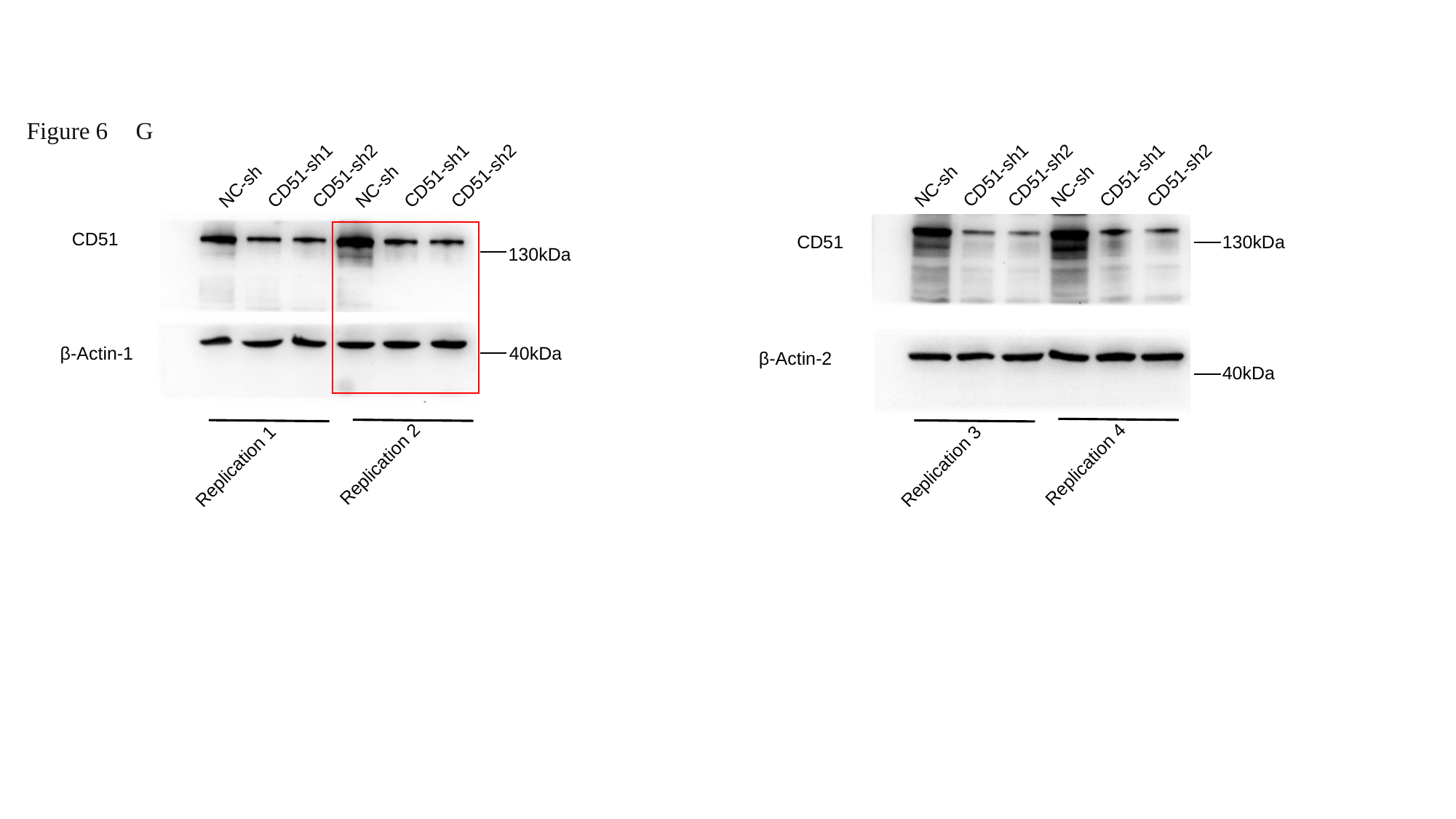

Figure 6	G
CD51-sh2
CD51-sh2
CD51-sh1
CD51-sh1
CD51-sh2
CD51-sh1
CD51-sh1
CD51-sh2
NC-sh
NC-sh
NC-sh
NC-sh
CD51
130kDa
β-Actin-1
40kDa
130kDa
CD51
β-Actin-2
40kDa
Replication 2
Replication 4
Replication 1
Replication 3

## Slide 4
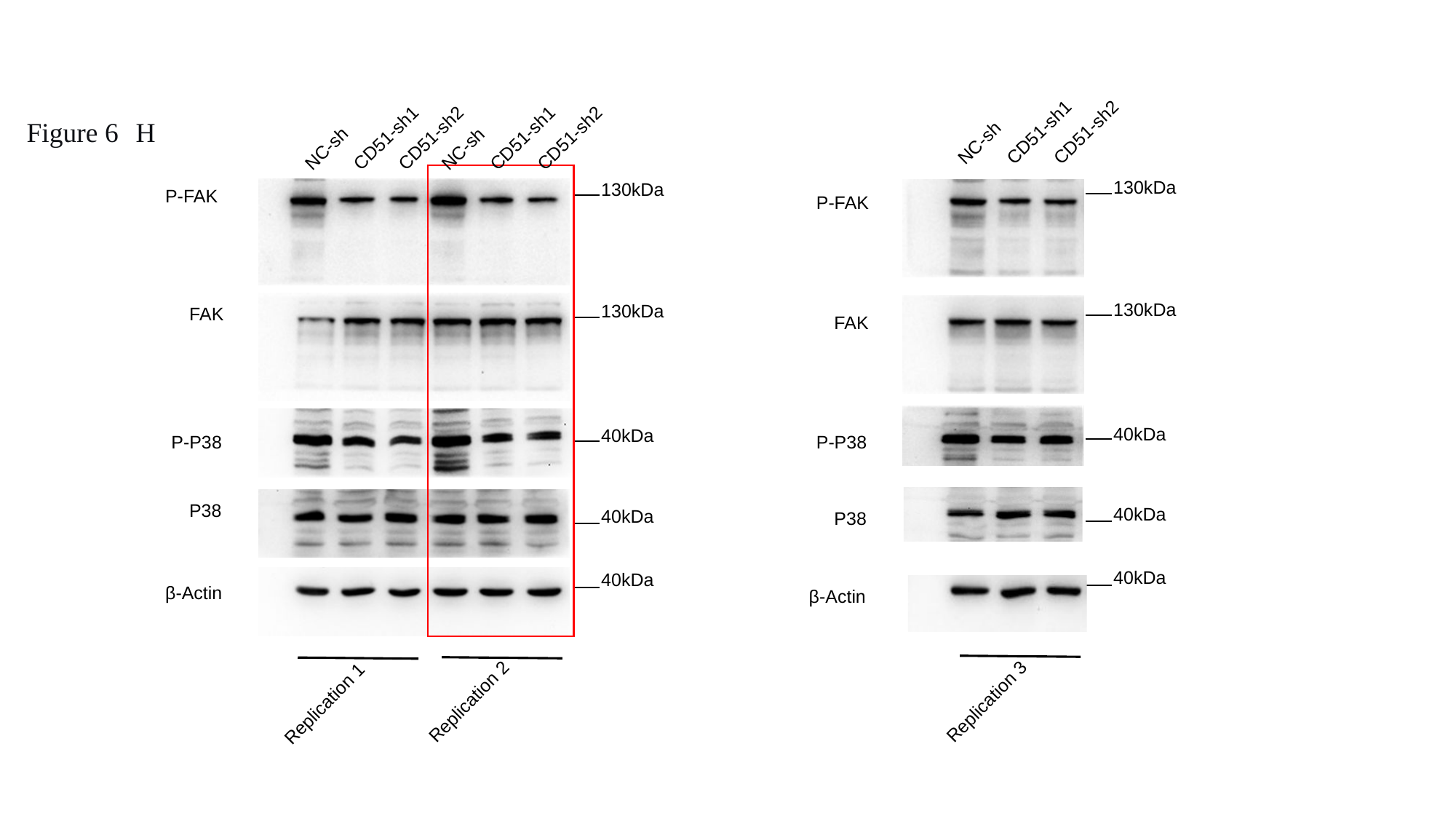

CD51-sh1
CD51-sh2
CD51-sh2
CD51-sh1
CD51-sh2
CD51-sh1
Figure 6	H
NC-sh
NC-sh
NC-sh
130kDa
P-FAK
130kDa
FAK
40kDa
P-P38
40kDa
P38
40kDa
β-Actin
130kDa
P-FAK
130kDa
FAK
40kDa
P-P38
P38
40kDa
40kDa
β-Actin
Replication 2
Replication 3
Replication 1

## Slide 5
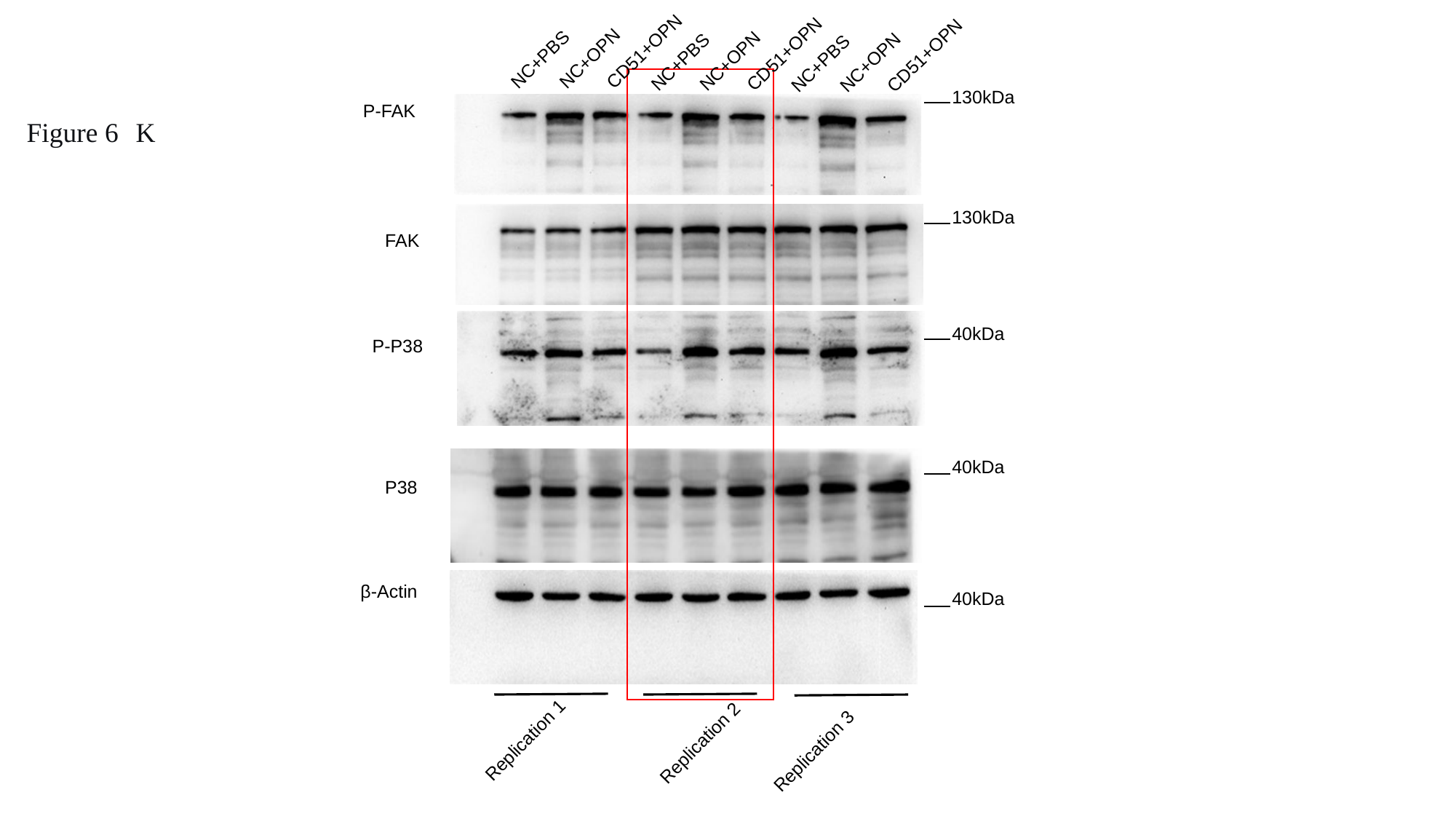

CD51+OPN
CD51+OPN
CD51+OPN
NC+OPN
NC+PBS
NC+OPN
NC+PBS
NC+OPN
NC+PBS
130kDa
P-FAK
130kDa
FAK
40kDa
P-P38
40kDa
P38
β-Actin
40kDa
Figure 6	K
Replication 1
Replication 2
Replication 3
